# The Effect of Cytochalasans on the Actin Cytoskeleton of Eukaryotic Cells and Preliminary Structure–Activity Relationships

**DOI:** 10.3390/biom9020073

**Published:** 2019-02-19

**Authors:** Robin Kretz, Lucile Wendt, Sarunyou Wongkanoun, J. Jennifer Luangsa-ard, Frank Surup, Soleiman E. Helaly, Sara R. Noumeur, Marc Stadler, Theresia E.B. Stradal

**Affiliations:** 1 Department of Microbial Drugs, Helmholtz Centre for Infection Research (HZI), Inhoffenstrasse 7, 38124 Braunschweig, Germany; kretzrob@hs-albsig.de (R.K.); lucile.wendt@helmholtz-hzi.de (L.W.); frank.surup@helmholtz-hzi.de (F.S.); soleiman.helaly@helmholtz-hzi.de (S.E.H.); noumeur_sara@yahoo.fr (S.R.N.); 2 University of Applied Sciences Albstadt-Sigmaringen, Faculty of Life Sciences, Anton-Günther-Strasse 51, 72488 Sigmaringen, Germany; 3 National Centre for Genetic Engineering and Biotechnology (BIOTEC), NSTDA, 113 Thailand Science Park, Phahonyothin Road, Klong Nueng Klong Luang, Pathum Thani 12120, Thailand; sarunyou.wong@biotec.or.th (S.W.); jajen@biotec.or.th (J.J.L.); 4 Department of Chemistry, Faculty of Science, Aswan University, 81528 Aswan, Egypt; 5 Department of Microbiology-Biochemistry, Faculty of Natural and Life Sciences, University of Batna 2, Batna 05000, Algeria; 6 Department of Cell Biology, Helmholtz Centre for Infection Research (HZI), Inhoffenstraße 7, 38124 Braunschweig, Germany

**Keywords:** actin cytoskeleton, Ascomycota, chromatography, secondary metabolites, structure elucidation, Xylariales

## Abstract

In our ongoing search for new bioactive fungal metabolites, two new cytochalasans were isolated from stromata of the hypoxylaceous ascomycete *Hypoxylon fragiforme*. Their structures were elucidated via high-resolution mass spectrometry (HR-MS) and nuclear magnetic resonance (NMR) spectroscopy. Together with 23 additional cytochalasans isolated from ascomata and mycelial cultures of different Ascomycota, they were tested on their ability to disrupt the actin cytoskeleton of mammal cells in a preliminary structure–activity relationship study. Out of all structural features, the presence of hydroxyl group at the C7 and C18 residues, as well as their stereochemistry, were determined as important factors affecting the potential to disrupt the actin cytoskeleton. Moreover, reversibility of the actin disrupting effects was tested, revealing no direct correlations between potency and reversibility in the tested compound group. Since the diverse bioactivity of cytochalasans is interesting for various applications in eukaryotes, the exact effect on eukaryotic cells will need to be determined, e.g., by follow-up studies involving medicinal chemistry and by inclusion of additional natural cytochalasans. The results are also discussed in relation to previous studies in the literature, including a recent report on the anti-Biofilm activities of essentially the same panel of compounds against the pathogenic bacterium, *Staphylococcus aureus*.

## 1. Introduction

Cytochalasans are a class of fungal metabolites, derived from mixed polyketide synthase/nonribosomal peptide synthetase (PKS/NRPS) biosynthesis that are widely distributed among the Ascomycota [1], and they occur particularly frequently in the genera of the order Xylariales [2]. Over the past decades, many of these compounds have been discovered in the course of natural product screening campaigns due to their prominent activities in biological systems and, in particular, their strong effects on eukaryotic cells [3]. Their biological activities have been attributed to their interactions with the actin cytoskeleton [4,5], even though this has so far only been established for a small portion of the representatives of this class of molecules [6]. The classic therapeutic indication for actin inhibitors is cancer, and some studies have been conducted in the past to evaluate the feasibility of obtaining a drug candidate based on this compound class [7], however, so far these activities have not been successful.

We have recently obtained a number of cytochalasans, including some new natural products, from different sources in the course of our ongoing search for novel bioactive fungal metabolites, and surprisingly found that several of them are able to significantly inhibit biofilm formation in the pathogenic bacterium, *Staphylococcus aureus* [8], while others did not show any activity. Since bacteria lack actin, this observation cannot be attributed to the known mechanism of action (MOA) of the compound class. Biofilm formation inhibitors may be of great utility for use in combination therapy with antibiotics since they may enhance the efficacy of the latter compounds [9,10]. On the other hand, an ideal biofilm inhibitor with therapeutic potential should neither possess activity against the pathogenic target microbes, in order to avoid upcoming resistance, nor should it be toxic to the cells of the host. We therefore decided to evaluate the panel of cytochalasans previously studied by Yuyama et al. [8], and some additional cytochalasans that have become available in our laboratory in the meantime, for their effects on mammalian cell lines using fluorescence microscopy, in order to find out more about the structure–activity relationships; our results are reported in the present paper.

## 2. Materials and Methods

### 2.1. Fungal Material

Stromata of *Hypoxylon fragiforme* were collected from *Fagus sylvatica* by L. Wendt in the vicinity of Braunschweig, Germany in 2017. A voucher specimen of the material is kept in the fungarium of M. Stadler at the Helmholtz Centre for Infection Research, Braunschweig, Germany (Acc. No. STMA18022). Stromata of *Daldinia* spp. were collected in Thailand, Chiang Mai Province, Ban Hua Thung community forest, on decaying wood by P. Srikitikulchai and S. Wongkanoun. Voucher specimens of the material are kept in the fungarium (BBH) and culture collection (BCC) of BIOTEC (Panthum Thani, Thailand). The stromata of both specimens were extracted as described previously [8].

The culture of *Preussia simillis* G22 was isolated from healthy roots of the medicinal plant Shrubby globularia (*Globularia alypum*) collected from Batna (Algeria) in March 2014 by S. R. Noumeur and was identified using the methods described previously [11]. The culture has been deposited with DSMZ, Braunschweig, Germany (designation No. DSM 32328) as well as in the culture collection of the Helmholtz Center for Infection Research (HZI).

### 2.2. Purification of the Compounds

Compounds **1** to **5**, **7**, **11** and **17** were purified from stromatal crude extracts by a preparative reversed-phase high-performance liquid chromatography (RP-HPLC) system (Gilson, Middleton, WI, USA) equipped with a GX-271 liquid handler, a diode array detector (DAD) 172 and a 305 and 306 pump. For the experiments, deionized water (solvent A), acetonitrile (ACN; solvent B, Avantor Performance Materials, Center Valley, PA, USA) and a VP Nucleodur C18ec (150 × 40 mm, 7 µm; Macherey-Nagel, Düren, Germany) column were used. The experiments were performed with a flow rate of 20 mL/min. To remove fatty acids and debris, the crude extracts were dissolved in acetonitrile and filtered through a Strata X-33 µm polymere reversed phase tube (Phenomenex, Aschaffenburg, Germany) prior to preparative liquid chromatography (LC) experiments. Fractions from preparative LC experiments were collected in round bottle flasks according to the ultraviolet (UV) absorption at 210 nm. Acetonitrile was removed from the fractions via evaporation in vacuo and the aqueous residues were frozen. An Alpha 1–4 LSC freeze dryer (Christ, Osterode, Germany) was used to remove the remaining water from the fractions.

Compounds **1**, **2**, and **17** were purified from stromata of *H. fragiforme* using the following gradient: The crude extracts were dissolved in ACN and the compounds purified by using an Agilent 1100 series preparative HPLC system (Agilent Technologies, Waldbronn, Germany). A Kromasil RP C18 (7mm, 250 × 25 mm; AkzoNobel, Mainz, Germany) and the mobile phase ACN and water was used (Milli-Q, Millipore, Schwalbach, Germany); flow rate 20 mL min^−1^. Isocratic conditions at 53% ACN were applied, followed by a linear gradient for 15 min to 67% ACN. Afterwards, another linear gradient to 100% ACN was applied. Fractions were combined according to UV adsorption at 220, 254 and 325 nm, solvents were evaporated, and liquid chromatography-mass spectrometry (LC-MS) analyses were performed. Fragiformin C (**1**) was eluted at *t*_R_ 10.2 min, fragiformin D (**2**) at *t*_R_ 8.9 min and compound (**17**) at *t*_R_ 9.5 min. The yields were ca. 2.7 mg of (**1**), 0.5 mg of (**2**) and 1 mg of (**17**) from 100 mg of crude extract.

Compound **3** was purified from stromata of *Daldinia sacchari* as described in [8].

Compounds **4**, **5**, **7** and **11** were purified from *P. simillis* DSM 32328 using the following conditions: The crude extracts were dissolved in methanol and purified by using an Agilent 1100 series preparative HPLC system (Agilent Technologies, Waldbronn, Germany); Kromasil RP C18 (7 mm, 250 × 25 mm; AkzoNobel, Mainz, Germany) column was used; mobile phase ACN and water (Milli-Q, Millipore, Schwalbach, Germany); flow rate 20 mL min^−1^. Isocratic conditions at 48% ACN and 52% water for 30 min were applied; fractions were combined according to UV adsorption at 220, 254 and 325 nm, solvents were evaporated, and LC-MS analyses were performed. Cytochalasin B (**4**) was eluted at *t*_R_ 10.2 min, deoxaphomin (**5**) at *t*_R_ 10.8 min, cytochalasin F (**7**) at *t*_R_ 11.7 min and cytochalasin Z2 (**11**) at *t*_R_ 13.5 min. The yields were ca. 17.8 mg of (**4**), 5 mg of (**5**), 0.8 mg of (7) and 1 mg of (11) from 256 mg of crude extract. Finally, compound **(6)** was purchased from Sigma-Aldrich (C8273, St. Louis, MO, USA).

The identification of the compounds was confirmed by high-resolution electrospray ionization mass spectrometry (HR-ESIMS) using the instrumental conditions described by Narmani et al. [12]. NMR spectra for structure elucidation were recorded with a Bruker Avance III 700 spectrometer with a 5 mm TCI cryoprobe (^1^H 700 MHz, ^13^C 175 MHz) and a Bruker Avance III 500 (^1^H 500 MHz, ^13^C 125 MHz) spectrometer (Bruker, Bremen, Germany). Chemical shifts δ were referenced to the solvents chloroform-d (^1^H, δ = 7.27 ppm; ^13^C, δ = 77 ppm; Sigma-Aldrich, St. Louis, MO, USA) and acetonitrile-d3 (^1^H, δ = 1.94 ppm; ^13^C, δ = 1.39 ppm; Sigma-Aldrich, St. Louis, MO, USA). Optical rotations were determined using a 241 MC polarimeter (Perkin Elmer, Waltham, MA, USA).

### 2.3. Spectral Data

#### 2.3.1. Fragiformin C

Colorless oil. [α]${}_{D}^{25}$ = +18.0 (c 1.0, AcN). ^1^H NMR (500 MHz, CDCl_3_): see Table 1; ^13^C NMR (125 MHz, CDCl_3_): see Table 1. HR-ESIMS *m/z* 434.2688 ([M + H]^+^, calcd for C_28_H_36_NO_3_ 434.2695).

#### 2.3.2. Fragiformin D

Colorless oil ([α]${}_{D}^{25}$ not determined for lack of material). ^1^H NMR (500 MHz, DMSO-*d*_6_): see Table 1; ^13^C NMR (125 MHz, DMSO-*d*_6_): see Table 1. HR-ESIMS *m/z* 450.2644 ([M + H]^+^, calcd for C_28_H_38_NO_4_ 450.2639).

### 2.4. Cytochalasans

All cytochalasans used are listed with their names in Table 2. For treatment of the cells, the cytochalasans were dissolved in DMSO (Carl Roth GmbH, Karlsruhe, Germany).

Cytochalasin B (**4**), F (**7**), Z2 (**11**) and deoxaphomin (**5**) were isolated from cultures of *P. simillis* as described above—cytochalasans “6” (**13**), “9” (**14**), “10” (**15**), “11” (**16**), 18-epi-cytochalasan 12 (**2**), L-696,474 (**9**), 21-O-deacyl-L-696,474 (**10**), 18-fragiformin A (**1**) and 18-epi-fragiformin B (**12**) were isolated previously from mycelial cultures of *H. fragiforme*, as described [8]. Compound **17** was isolated from *Daldinia* spp. as described in 2.2. Cytochalasin H (**8**), 19,20-epoxycytochalasin C, 19,20-epoxycytochalasin D, 19,20-epoxycytochalasin N, 18-deoxy-19,20-epoxycytochalasin Q (**18–21**) and the phenochalasins C (**22**) and D (**23**) were isolated from *Daldinia* spp. by RP-HPLC as described in [8]. Chaetoglobosins were isolated previously from *Ilyuha vitellina* [13]. The organism “*Hypoxylon kretzschmarioides”*, from which the phenochalasins C and D were derived [8], has meanwhile been subjected to a taxonomic study that resulted in its transfer to the genus *Daldinia* and the herbarium specimen represents the epitype of *Daldinia kretzschmarioides*, comb. nov. [14].

### 2.5. Cell Culture

U2OS, a human osteosarcoma cell line [ATCC HTB-96] was cultured in Dulbecco’s modified minimum essential medium (DMEM, Life Technologies, Carlsbad, CA, USA) containing 10% fetal bovine serum, 1% l-glutamine, 1% minimum essential medium nonessential amino acids (MEM NEAA) and 1% sodium-pyruvate (Life Technologies, Carlsbad, CA, USA) at 36 °C and 5% CO_2_.

### 2.6. Cytochalasan Treatment

For cytochalasan treatment, the cells were grown on glass coverslips. Prior to cell growth, the coverslips were coated with 25 µg/mL fibronectin in phosphate buffered saline (PBS) for one hour. Cytochalasans were applied at a concentration of 1 and 5 µg/mL for 1 h. Washout experiments were conducted by exchanging the cytochalasan-containing medium with DMEM after incubation time and incubating for another hour. Further details can be found in the Supplementary Information.

### 2.7. Immunofluorescence

Treated cells were fixed with 4% paraformaldehyde (AppliChem, Darmstadt, Germany) in PBS for 20 min at room temperature. Fixed cells were washed with PBS, permeabilized with 0.1% Trition X-100 (Bio-Rad Laboratories, Hercules, CA, USA) in PBS for 1 min at room temperature and again transferred to PBS. The actin cytoskeleton was stained using fluorescently labelled phalloidin ATTO 594 (1:200 ATTO-Tec, Siegen, Germany) in PBS for 1 h. The cover slips were mounted in Prolong Diamond antifade mountant with DAPI (inVitrogen, Carlsbad, CA, USA) to stain the nucleus. Pictures were taken with a Axio Vert 135 TV inverse microscope with phase contrast and CoolSnap 4k camera (Zeiss, Oberkochen, Germany). Pictures were processed using Image J (NIH, Bethesda, MD, USA).

## 3. Results and Discussion

### 3.1. Structure Elucidation of the New Compounds

A fruiting body extract of *H. fragiforme* was fractionated by reversed-phase HPLC and provided the new metabolites **1** and **2** as colorless oils. For **1,** the molecular formula C_28_H_35_NO_3_ was deduced based on its [M + H]^+^ and [M+Na]^+^ peaks at *m/z* 434.2688 and 456.2502 in the HRESIMS spectrum, implying 12 degrees of unsaturation. ^1^H and HSQC NMR spectra revealed the presence of four methyls, three methylenes, and seven olefinic (two with dual intensity) as well as six aliphatic methines. In addition, the ^13^C spectrum specified a conjugated ketone, an amide carbon, and four further carbons devoid of bound protons. Subsequently, spin systems were constructed by ^1^H,^1^H COSY and TOCSY correlations, which were connected by HMBC correlations, to form a cytochalasan skeleton (Figure S1). The closest structural relative for the planar structure of **1** is fragiformin A, the 18-hydroxyl derivative of **1** [15]. The stereochemistry of **1** was assigned by ROESY data: ROESY correlations between 13–H and 20–H as well as 14–H and 19–H supported the characteristic conformation described for the eleven-membered ring system [15]. ROESY correlations between 23–H_3_ and 20–H, located below the molecular main plain, in addition to those between 18–H and 16–H, located above the molecular main plain, confirm the downwards orientation of 23–H_3_ and upwards orientation of 18–H and thus an 18*S* configuration. We propose the trivial name fragiformin C for compound **1**, whose systematic IUPAC name is (7*S*,13*E*,16*S*,18*S*,19*E*)-16,18-dimethyl-7-hydroxy-10-phenyl-[11]-cytochalasa-5,13,19-triene 1,21-dione [16].

Metabolite **2**, isolated from the stromatal (fruiting body) extract of *H. fragiforme,* was analyzed for its molecular formula C_28_H_35_NO_4_ by HRESIMS, indicating a formal addition of an oxygen atom compared to **1**. The NMR data of **2** showed a similarity to those of **1** for specifying the cytochalasin scaffold. An analysis of ^1^H, ^13^C and HSQC data identified the key differences as the replacement of the C–5/C–6 double bond by a methine and an oxygenated carbon devoid of bound protons, and the replacement of methine C–18 by oxygenation. Consequently, **2** was elucidated as 16,18-dimethyl-6,7epoxy-18-hydroxy-10-phenyl-[11]-cytochalasa–13,19-diene-1,21-dione, which has been described previously [17]. However, a careful examination of ^13^C chemical shifts revealed significant differences, specifically for C–17 and C–23. Therefore, the configuration of **2** was examined by ROESY data. Analogously to **1**, ROESY correlations between 23–H_3_ and 20–H and between 18–H and 16–H confirmed the downwards orientation of 23–H_3_ and the upwards orientation of 18–H, respectively. Consequently, compound **2** with its 18*R* configuration is the 18-epimer of the known compound **17** isolated from a *Daldinia* species [18], which was later identified as *D. eschscholtzii* [19]. Its systematic name is (7*S*,13*E*,16*S*,18*R*,19*E*)- 16,18-dimethyl-6,7epoxy-18-hydroxy-10-phenyl-[11]-cytochalasa–13,19-diene-1,21-dione [13], and here we propose the trivial name fragiformin D for the compound.

Interestingly, the two novel cytochalasins that we report in this study were actually discovered in the course of another project aimed at the identification of complex azaphilone pigments that were detected in the stromata of carbonized, fossil *H. fragiforme* originating from excavations in France [20]. However, they were not detected in the ancient specimens but only in the recently collected reference material that was used to isolate the metabolites in sufficient quantities for structure elucidation. The producer organism is actually the type species of the recently resurrected family Hypoxylaceae [21] and belongs to the most frequently encountered macromycetes in the Northern hemisphere.

Cytochalasans B (**4**), F (**7**), Z2 (**11**) and deoxaphomin (**5**) were concurrently isolated from the endophytic fungus *Preussia similis* DSM 32328, and their structures were identified by comparing their ^1^H and ^13^C chemical shifts as well as the HRMS data to those reported in the literature (references [22,23] for compound **4**; reference [23] for compound **5**; references [24,25] for compound **7**; reference [26] for compound **11**). An authentic sample of commercially available cytochalasin B was used for comparison, and the NMR data of the isolated cytochalasin B (**4**) were identical with the commercial sample.

**Figure 1:**
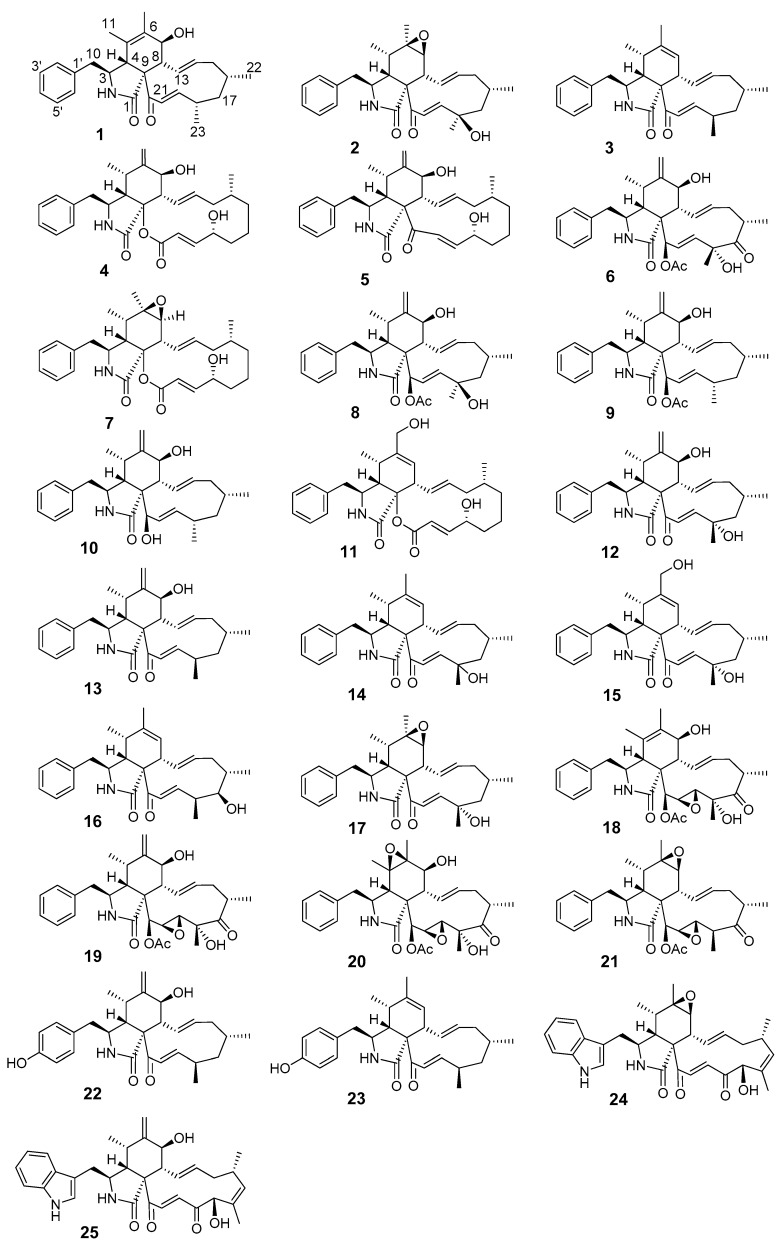
Chemical structures of cytochalasans employed in this study.

Further known cytochalasans (Table 2, Figure 1) were isolated from other Sordariomycetes species and identified comparing the ^1^H and ^13^C chemical shifts and the HRMS data to those reported in the literature as previously described (see Table 2).

It is interesting to compare the chemical shift of methyls C–22 and C–23 of **1** and **2** to those of compounds that are epimeric at C–18, but otherwise possess an identical carbon backbone, such as **2** and **17**. Whereas the chemical shifts δ_C_ of C–22 are nearly identical in all cases (**9**: 25.3; **10**: 25.2; **3**: 26.1; **13**: 26.2), the C–23 chemical shifts differ significantly (**9**: 22.1; **10**: 22.4; **3**: 17.5; **13**: 17.6).

The same fact was observed for compounds that are oxidized at C–18, such as **12** and its 18-epimer, fragiformin B (see NMR data in reference [15] for comparison); C–23 is significantly shifted downfield in fragiformin B (δ_C_ 30.2) compared to **12** (δ_C_ 25.7). Consequently, the chemical shift of methyl CH_3_-23 might be assessed as an indicator for the variable stereochemistry at C–18. The chemical structures of all compounds tested are shown in Figure 1.

### 3.2. Effects of Cytochalasans on Cell Cultures

The effects of the compounds on the actin cytoskeleton of mammalian cells were analyzed by fluorescence microscopy upon staining with fluorescently labelled phalloidin to stain for filamentous actin (F-actin) and with 4′,6-diamidino-2-phenylindole (DAPI) to stain for nuclear DNA. To enable comparability between the compounds, the effects on cellular F-actin structures were analyzed at two different concentrations (and at two different exposure times, not shown) and classified using stepwise gradations from “**+++**” to “**-**“ (see Table 2) as follows: compounds leading to complete disruption of the actin cytoskeleton at a concentration of 1 µg/mL were marked with “**+++**”; compounds leading to complete disruption of the cytoskeleton at concentrations of 5 µg/mL were classified as “**++**”; compounds showing an incomplete disruption of the cytoskeleton at 5 µg/mL were categorized as “**+**”; and finally, compounds that did not affect the actin cytoskeleton at all in this study were classified as “**-**“.

**Figure 2:**
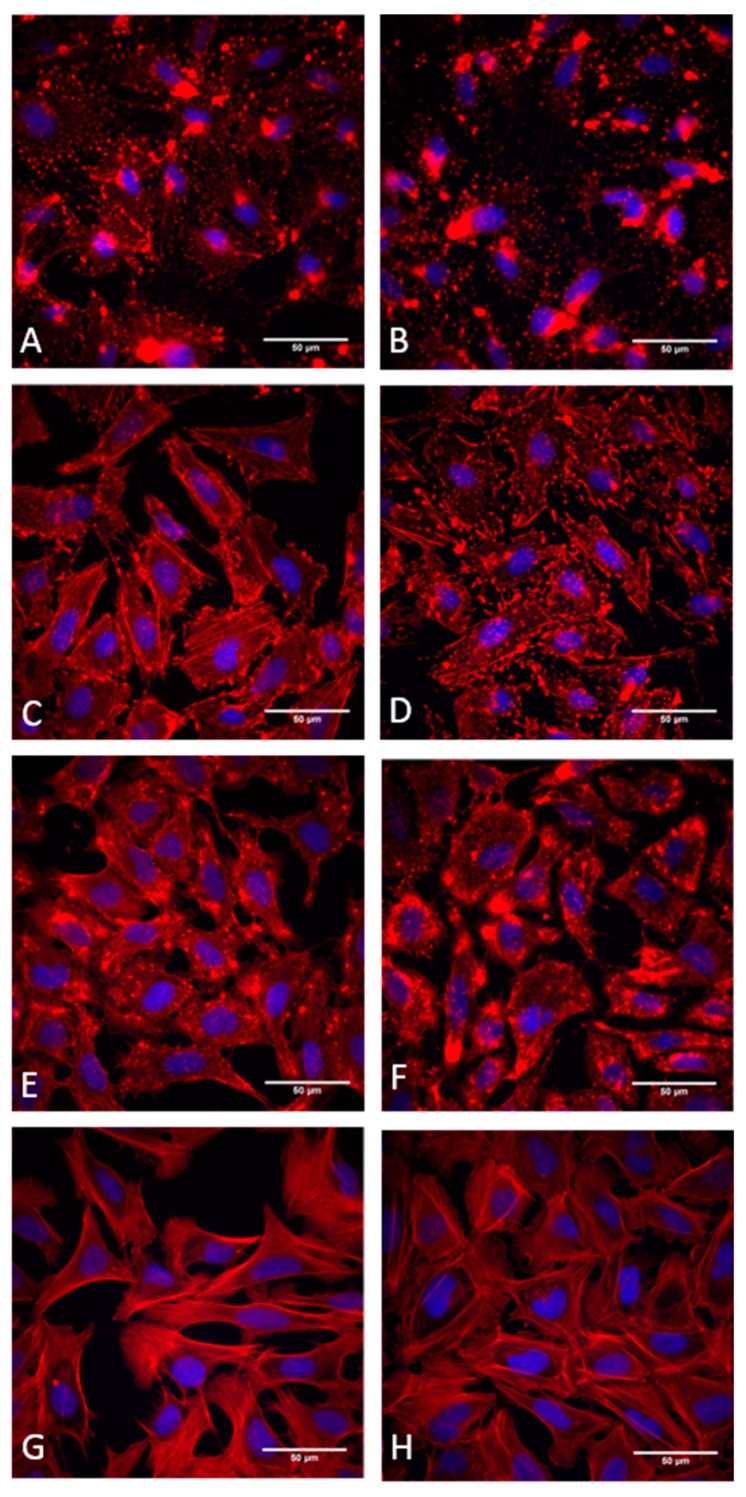
Immunofluorescence staining with phalloidin of U2OS cells treated with cytochalasans. (**A**) Treated with 1 µM cytochalasin H (**8**). (**B**) Treated with 5 µM cytochalasin H. (**C**) Treated with 1 µM cytochalasin B (**4**). (**D**) Treated with 5 µM cytochalasin B. (**E**) treated with 1 µM chaetoglobosin D (**25**). (**F**) Treated with 5 µM chaetoglobosin D. (**G**) Treated with 5 µM DMSO (negative control). (**H**) Washout after treatment with 5 µM cytochalasin H.

The reversibility of cytochalasan-mediated effects was assessed in a 1 h-washout experiment and classified as follows: full recovery of actin structures was categorized as reversible “**+**”; partial recovery as partially reversible “**+/-**“; and, when disruption of the actin cytoskeleton remained severe even after washout, the effects were classified as irreversible “**-**“. Results of these experiments are summarized in Table 2 and representative examples of fluorescently labelled cells are displayed in Figure 2.

Of all the cytochalasans, cytochalasin B (**4**) and its effect on the actin cytoskeleton has been characterized best, as revealed by various reports in the literature [6,27,28]. Therefore, this metabolite was used as the reference compound in this study. Notably, most cytochalasans tested here showed characteristic phenotypic changes of the actin cytoskeleton upon treatment. However, the extent of actin cytoskeleton disruption was highly variable. Less severe effects (e.g., compounds **7** and **17**) showed small patches of aggregated actin and rearrangement but no loss of stress fibers. In contrast, cells treated with more potent compounds showed complete actin disruption and the formation of dense, asterisk-like F-actin aggregates (e.g., compounds **2** and **18**).

These phenotypic changes of the actin cytoskeleton have been reported previously in several studies [6,29,30]. Treatment with the two chaetoglobosins studied, which—like the majority of the compounds used in our study, were evaluated here for the first time—resulted in a different phenotype, which also showed actin aggregation. However, this was not associated with a significant loss of the pre-existing actin filaments.

To reveal structural features of cytochalasans that correlate with the potency of the compounds to disrupt the actin cytoskeleton, we performed a meta-analysis and inspected the chemical structures of the different potency groups in depth. Our analysis revealed common features among the grouped compounds. Hydroxyl groups at C7 and C18 were most prominent in the “**+++**” group, with each compound showing at least one of these. In the “**-**“ group, both functional groups were completely absent, emphasizing the importance of these structural feature for actin disruption. The importance of the C7 hydroxyl group also was reported for anticancer activity [27], which may be related to the effect on the actin cytoskeleton. Most striking are differences in the activities of structurally closely related compounds, such as phenochalasins C (**22**) and D (**23**), and the compound **17** and fragiformin D (**2**). Phenochalasin D (**23**) differs from phenochalasin C (**22**) by the addition of a hydroxyl group at C7, as well as a methyl group instead of an exocyclic double bond at C6. However, the cells showed strong actin disruption (“**++**”) upon treatment with phenochalasin D, but no actin disruption upon treatment with phenochalasin C, highlighting the importance of the C7 hydroxyl group for activity once more (see Table S1).

Comparison of compound **17** (“**+**”) and fragiformin D (**2**) (“**+++**”) revealed the importance of stereochemistry of functional groups for actin disruption activity (see Table S1). Also, the stereochemistry of the C18 group seems to affect the binding affinity towards actin, since fragiformin D showed an irreversible, and compound **17** a reversible, actin disruption. Also, a high abundance of compounds showing an acetylated C21 residue is located in the “**+++**” group, indicating a possible correlation of this feature with high actin disruption capacity. Lastly, higher conformational freedom of the macrocycle of cytochalasans was reported to positively affect the anticancer activity of cytochalasans, whereas the size of the macrocycle did not [31]. Here, no evidence for a correlation between conformational freedom of the macrocycle and actin disruption capacity was found. However, all highly potent compounds (“**+++**”) with the exception of deoxaphomin (**5**), showed a 11-membered macrocycle, which is the smallest possible macrocycle reported for cytochalasans. In support of this notion, higher activity of cytochalasans with 11-membered macrocycles compared to 14-membered macrocycles has been reported previously [32]. Comparing the trait of reversibility of the compounds, none of the different potency groups correlated with being reversible or irreversible. This indicates that the reversibility of cytochalasan effects is not structurally linked to their potency concerning actin disruption. However, all compounds showing irreversible effects on actin filaments carry only few functional groups in addition to hydroxylated C–7 residue. Also, none of these compounds is acetylated at C–21. The absence of additional functional groups in the irreversible compounds groups indicates that, in general, the small size of the compounds contributes to irreversibility.

As mentioned in the introduction, we recently have studied a largely overlapping panel of cytochalasans that were examined here for their effect on the biofilm formation of *S. aureus* [8] and found likewise that some of the tested molecules had rather strong inhibitory effects, while others were completely inactive in this bioassay. According to preliminary results, the degree of biofilm inhibition can be influenced by, e.g., the presence of an isomeric double bond in the macrocycle, the degree of acetylation of the hydroxyl groups, or the presence of a phenol at C4′ phenyl group, as well as epoxy groups in the macrocyclic ring. Notably, these results were mostly deduced from pairwise comparison of closely related congeners, and not in the classical manner for establishing structure–activity relationships, i.e., by semisynthetic modification of the same basic structure. Therefore, the results must be regarded as tentative and can only guide medicinal chemists in a future concise optimization program to find the best inhibitor. Interestingly, despite the two traits (biofilm inhibition vs. actin modulation) probably having different MOA, there are three compounds, i.e., L-696,474 (**9**), epoxycytochalasin C (**18**) and compound **12**, which show strong biofilm inhibition as well as strong actin disruption. However, further correlations between the two traits could not be determined. Recently, it was shown that cytochalasans can cause either cytotoxic or cytostatic effects in human cancer cells [31]. Those compounds that could not be removed from the actin filaments, indicating that they irreversibly inhibited actin-related processes in cells and ultimately will prolong apoptotic signaling, may constitute good candidates for development of anticancer drugs by a rational, medicinal chemistry-driven optimization program. On the other hand, compounds **14** and **15** from *D. eschscholtzii*, and in particular the chaetoglobosins, might serve as initial structures for development of biofilm inhibitors if their toxicity could be further reduced by semisynthesis, or even total synthesis, because they showed relatively low potency against actin but were among the strongest inhibitors of biofilm in *S. aureus* in our previous study. However, this topic will have to be addressed in future studies.

## 4. Conclusions

This study described the isolation of two new cytochalasans from *H. fragiforme*, adding to the growing the number of cytochalasans. Recently, with the discovery of cytotoxic, cytostatic, antiviral and anti-biofilm activities of cytochalasans, interest in using these compounds as therapeutic agents is reemerging. However, the use in human therapy requires excellent knowledge and in-depth characterization of the molecular effects caused by these fungal metabolites. The aim of this study was to systematically analyze the effects of cytochalasans on the actin cytoskeleton in vivo and link these to structural features of the compounds. This meta-analysis aims at enabling the prediction of the molecular effects of novel cytochalasan derivatives. Our data strongly suggest that the presence of C–7 and C–18 hydroxylation is correlated with high potency to disrupt the actin cytoskeleton of eukaryotes, very likely related to their cytotoxic and cytostatic activities. Also, we show here that the capability of the compounds to disrupt the actin cytoskeleton does not directly correlate with the reversibility of this effect, which in turn seems to be connected to the compound size. This important information helps to understand the effects of these metabolites and paves the way for future approaches to synthesize compounds with the desired features but lacking unwanted activities. We therefore propose that certain compounds should be isolated from the available fungal strains in larger quantities and subjected to a microderivatization program, or obtained by total synthesis in larger quantities and finally subjected to a broad biological characterization including the evaluation of their biological activities against mammalian cells, viruses and pathogenic microbes. This endeavor would afford substantial capacities for biotechnological production of the molecules and medicinal chemistry.

## Figures and Tables

**Table 1:** Nuclear magnetic resonance (NMR) spectroscopic data for fragiformins C (**1**) and D (**2**).

	1 ^a^		2 ^b^	
	δ_C_, mult.	δ_H_, mult.	δ_C_, mult.	δ_H_, mult.
**1**	174.2, C		173.2, C	
**2**		5.56, br s		8.35, br s
**3**	59.1, CH	3.41, m	52.5, CH	3.59, m
**4**	47.0, CH	3.64, br s	43.9, CH	3.01, br d (6.3)
**5**	126.2, C		35.3, CH	1.45, m
**6**	131.6, C		57.5, C	
**7**	69.5, CH	4.08, d (9.5)	61.7, CH	2.78, d (5.8)
		OH: 1.26, br s		
**8**	53.5, CH	2.09, m	49, CH	1.94, m
**9**	62.6, C		65.5, C	
**10**	42.8, CH_2_	2.69, dd (13.4, 7.5)2.63, dd (13.4, 7.5)	43.3, CH_2_	2.71, dd (13.0, 4.1)
				2.19, dd (13.0, 9.2)
**11**	17.1, CH_3_	1.44, s	12.1, CH_3_	0.56, d (7.2)
**12**	14.1, CH_3_	1.70, s	19.1, CH_3_	1.12, s
**13**	127.2, CH	6.04, ddd (15.7, 10.1, 1.0)	127.3, CH	5.85, ddd (15.5, 9.6, 1.0)
**14**	138.6, CH	5.20, ddd (15.7, 10.9, 4.8)	135.4, CH	4.94, ddd (15.5, 10.8, 4.5)
**15**	42.6, CH_2_	2.01, m	42.5, CH_2_	1.93, m
		1.84, ddd (12.0, 11.0, 10.9)		1.69, m
**16**	32.7, CH	1.33, m	28.3, CH	1.61, m
**17**	49.2, CH_2_	1.70, m	53.9, CH_2_	1.69, m
		1.50, dt (13.8, 3.8)		1.52, m
**18**	34.8, CH	2.44, m	73, C	OH: 4.83, s
**19**	155.4, CH	7.14, dd (16.4, 7.2)	155.4, CH	6.58, d (16.5)
**20**	130.6, CH	7.01, br d (16.4)	129.2, CH	6.73, d (16.5)
**21**	196.7, C		195.5, C	
**22**	25.0, CH_3_	1.03, d (7.0)	26.2, CH_3_	0.98, d (6.8)
**23**	20.8, CH_3_	1.10, d (6.9)	30, CH_3_	1.21, s
**1′**	137.4, C		136.8, C	
**2′/6′**	129.2, CH	7.21, br d (7.8)	129.7, CH	7.18, br d (7.7)
**3′/5′**	128.7, CH	7.33, br t (7.8)	128.2, CH	7.29, br t (7.7)
**4′**	126. 9, CH	7.25, br t (7.8)	126.5, CH	7.21, br t (7.7)

^a^ 700 Mhz for ^1^H, 175 MHz for ^13^C in CHCl_3_-*d*, ^b^ 500 Mhz for ^1^H, 125 MHz for ^13^C in DMSO-*d*_6_.

**Table 2:** Effects of cytochalasans on mammalian cells and against biofilms of *Staphylococcus aureus*. ***Actin disruption***: **+++** complete disruption at 1 µg/mL, **++** complete disruption at 5 µg/mL, **+** incomplete disruption at 5 µg/mL, **-** no disruption; Reversibility: **+** reversible effect, **+/-** partially reversible effect, **-** irreversible; **nd**: not determined because it was not active in the first place. ***Anti-Biofilm activity***: activities taken from the study by Yuyama et al. [8]; **nt**: compound not tested, due to insufficient amounts available or apparent instability.

	Trivial Name	Actin Disruption	Reversible	Anti-Biofilm [8]	Biological source
**1**	Fragiformin C	**+**	**+/-**	**nd**	*Hypoxylon fragiforme* (this study)
**2**	Fragiformin D	**+++**	**-**	**nd**	*H. fragiforme* (this study)
**3**	Saccalasin A	**-**	**nt**	**+**	*Daldinia sacchari* [12]
**4**	Cytochalasin B	**++**	**+**	***-***	*Preussia similis* (this study)
**5**	Deoxaphomin	**+++**	**-**	**+**	*P. similis* (this study)
**6**	Cytochalasin D	**+++**	**+/-**	**-**	*Zygosporium mansorii* (Sigma)
**7**	Cytochalasin F	**+**	**+**	**nd**	*P. similis* (this study)
**8**	Cytochalasin H	**+++**	**+**	**-**	*H. fragiforme* [8]
**9**	L-696,474	**+++**	**+**	**++**	*H. fragiforme* [8]
**10**	21-O-Deacyl-L-696,474	**+++**	**+**	**+**	*H. fragiforme* [8]
**11**	Cytochalasin Z2	**+**	**+**	**nd**	*P. similis* (this study)
**12**	“Cytochalasin 6” [16]	**+++**	**-**	**+++**	*D. eschscholtzii* [8]
**13**	“Cytochalasin 9” [16]	**++**	**-**	**-**	*D. eschscholtzii* [8]
**14**	“Cytochalasin 10” [17]	**+**	**+/-**	**+++**	*D. eschscholtzii* [8]
**15**	“Cytochalasin 11” [17]}	**+**	**+/-**	**+++**	*D. eschscholtzii* [8]
**16**	“Cytochalasin 12” [18]	**-**	**nt**	**+**	*D. eschscholtzii* [8]
**17**	New Cytochalasin	**+**	**+/-**	**nd**	*D. eschscholtzii* [8]
**18**	19,20-Epoxycytochalasin C	**+++**	**+**	**++**	*Rosellinia rickii* [8]
**19**	19,20-Epoxycytochalasin D	**+++**	**+/-**	**-**	*R. rickii* [8]
**20**	19,20-Epoxycytochalasin N	**+**	**+**	**-**	*R. rickii* [8]
**21**	18-Deoxy-19,20-Epoxy-cytochalasin Q	**++**	**+**	**-**	*R. rickii* [8]
**22**	Phenochalasin C	**++**	**-**	**+**	*H./D. kretzschmarioides* [8]
**23**	Phenochalasin D	**-**	**nt**	**++**	*H./D. kretzschmarioides* [8]
**24**	Chaetoglobosin A	**+**	**+**	**+++**	*Ijuhya vitellina* [13]
**25**	Chaetoglobosin D	**++**	**-**	**nd**	*Il. vitellina* [13]

## Supplementary Materials

Supplementary material can be found at http://www.mdpi.com/2218-273X/9/2/73/s1. Figures S1 and S2: COSY and TOCSY (blue arrows), HMBC (green arrows) and ROESY (violet arrows) correlations indicating the structures of fragiformin C (1) and fragiformin D (2). Figure S3: HPLC-HRESIMS data of 1. Figure S4: ^1^H NMR spectrum (700 MHz, CDCl3) of 1. Figure S5: ^13^C NMR spectrum (175 MHz, CDCl3) of 1. Figure S6: COSY NMR spectrum (700 MHz, CDCl3) 1. Figure S7: ROESY NMR spectrum (700 MHz, CDCl3) 1. Figure S8: HSQC NMR spectrum (700 MHz, CDCl3) 1. Figure S9: HMBC NMR spectrum (700 MHz, CDCl3) 1. Figure S10: HPLC-HRESIMS data of 2. Figure S11: ^1^H NMR spectrum (500 MHz, DMSO-d6) of 2. Figure S12: ^13^C NMR spectrum (125 MHz, DMSO-d6) of 2. Figure S13: COSY NMR spectrum (500 MHz, DMSO-d6) of 2. Figure S14: ROESY NMR spectrum (500 MHz, DMSO-d6) of 2. Figure S15: HSQC NMR spectrum (500 MHz, DMSO-d6) of 2. Figure S16: HMBC NMR spectrum (500 MHz, DMSO-d6) of 2. Figure S17: ^1^H NMR spectrum (500 MHz, CDCl3) of 17. Figure S18: ^13^C NMR spectrum (125 MHz, CDCl3) of 17. Figure S19: ^1^H NMR spectrum (500 MHz, Acetone-d6) of cytochalasin B (4). Figure S20: ^13^C NMR spectrum (500 MHz, Acetone-d6) of cytochalasin B (4). Figure S21: HSQC NMR spectrum (500 MHz, Acetone-d6) of cytochalasin B (4). Figure S22: ^1^H NMR spectrum (500 MHz, Acetone-d6) of deoxaphomin (5). Figure S23: ^13^C NMR spectrum (500 MHz, Acetone-d6) of deoxaphomin (5). Figure S24: ^1^H NMR spectrum (500 MHz, Acetone-d6) of cytochalasin F (7). Figure S25: ^13^C NMR spectrum (500 MHz, Acetone-d6) of cytochalasin F (7). Figure S26: ^1^H NMR spectrum (500 MHz, Acetone-d6) of cytochalasin Z2 (11). Figure S27: ^13^C NMR spectrum (500 MHz, Acetone-d6) of cytochalasin Z2 (11). Table S1: Cytochalasan treatment of U2OS cells.
