# Supplementary material for: The Effect of Cytochalasans on the Actin Cytoskeleton of Eukaryotic Cells and Preliminary Structure–Activity Relationships
Source: Biomolecules. 2019 Feb 19;9(2):73. doi: 10.3390/biom9020073 (PMC6406453; doi:10.3390/biom9020073)
Supplement: Supplementary file 1 [file biomolecules-09-00073-s001.pdf]

Article

The effect of Cytochalasans on the Actin Cytoskeleton of Eukaryotic Cells and Preliminary Structure-Activity Relationships

Robin Kretz [1,2], Lucile Wendt [1], Sarunyou Wongkanoun [3], J. Jennifer Luangsa-ard [3] Frank Surup [1], Soleiman E. Helaly [1,4], Sara R. Noumeur [1,5], Marc Stadler [1,*] and Theresia Stradal[6]

1 Department of Microbial Drugs, Helmholtz Centre for Infection Research (HZI), Inhoffenstrasse 7, 38124 Braunschweig, Germany; kretz@hs-albsig.de (R.K.); lucile.wendt@helmholtz-hzi.de (L.W.); frank.surup@helmholtz-hzi.de (F.S.); soleiman.helaly@helmholtz-hzi.de (S.H.)
2 University of Applied Sciences Albstadt-Sigmaringen, Anton-Günther-Strasse 51, 72488 Sigmaringen, Germany
3 National Centre for Genetic Engineering and Biotechnology (BIOTEC), NSTDA, 113 Thailand Science Park, Phahonyothin Road, Klong Nueng Klong Luang, Pathum Thani 12120 Thailand; sarunyou.wong@biotec.or.th (S.W.); jajen@biotec.or.th (J.J.L.)
4 Department of Chemistry, Faculty of Science, Aswan University, 81528 Aswan, Egypt
5 Department of Microbiology-Biochemistry, Faculty of Natural and life sciences, University of Batna 2, 05000 Batna, Algeria
6 Department of Cell Biology, Helmholtz Centre for Infection Research and German Centre for Infection Research (DZIF), partner site Hannover/Braunschweig, Inhoffenstraße 7, 38124 Braunschweig, Germany; theresia.stradal@helmholtz-hzi.de (T.S.)

* Correspondence: theresia.stradal@helmholtz-hzi.de; marc.stadler@helmholtz-hzi.de; Tel.: +49-531-6181-4240; Fax: +49-531-6181-9499

------------------------Supporting Information-----------------------

1

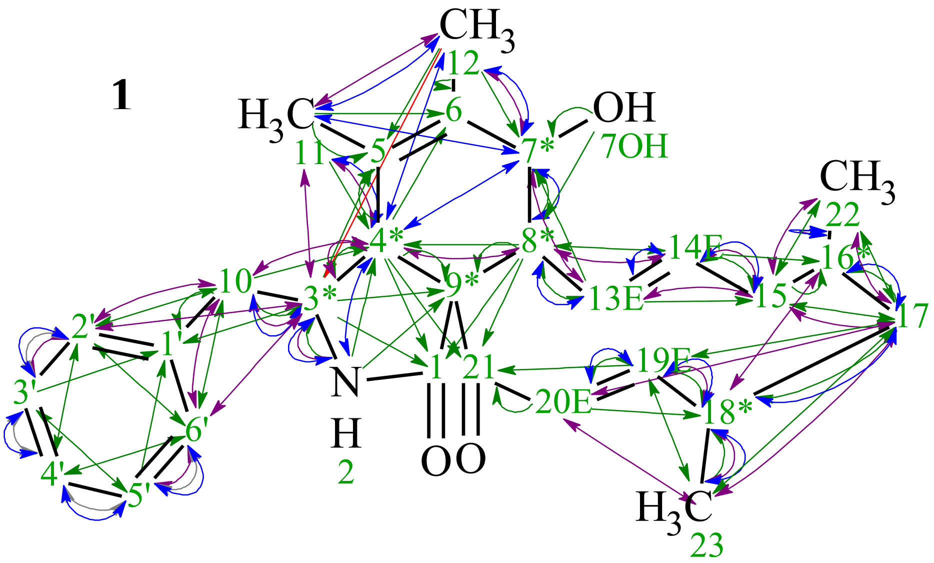

2

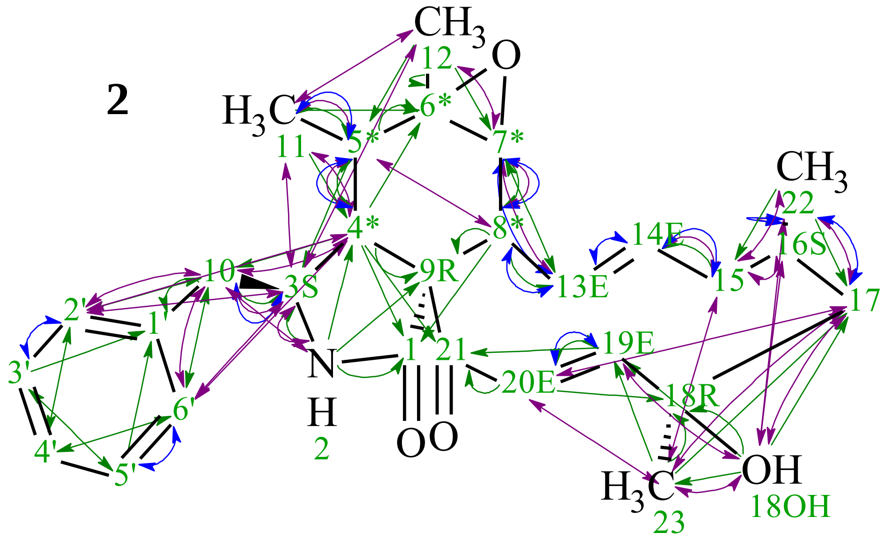

Figures S1 & S2. COSY and TOCSY (blue arrows), HMBC (green arrows) and ROESY (violet arrows) correlations indicating the structures of **1** and **2**.

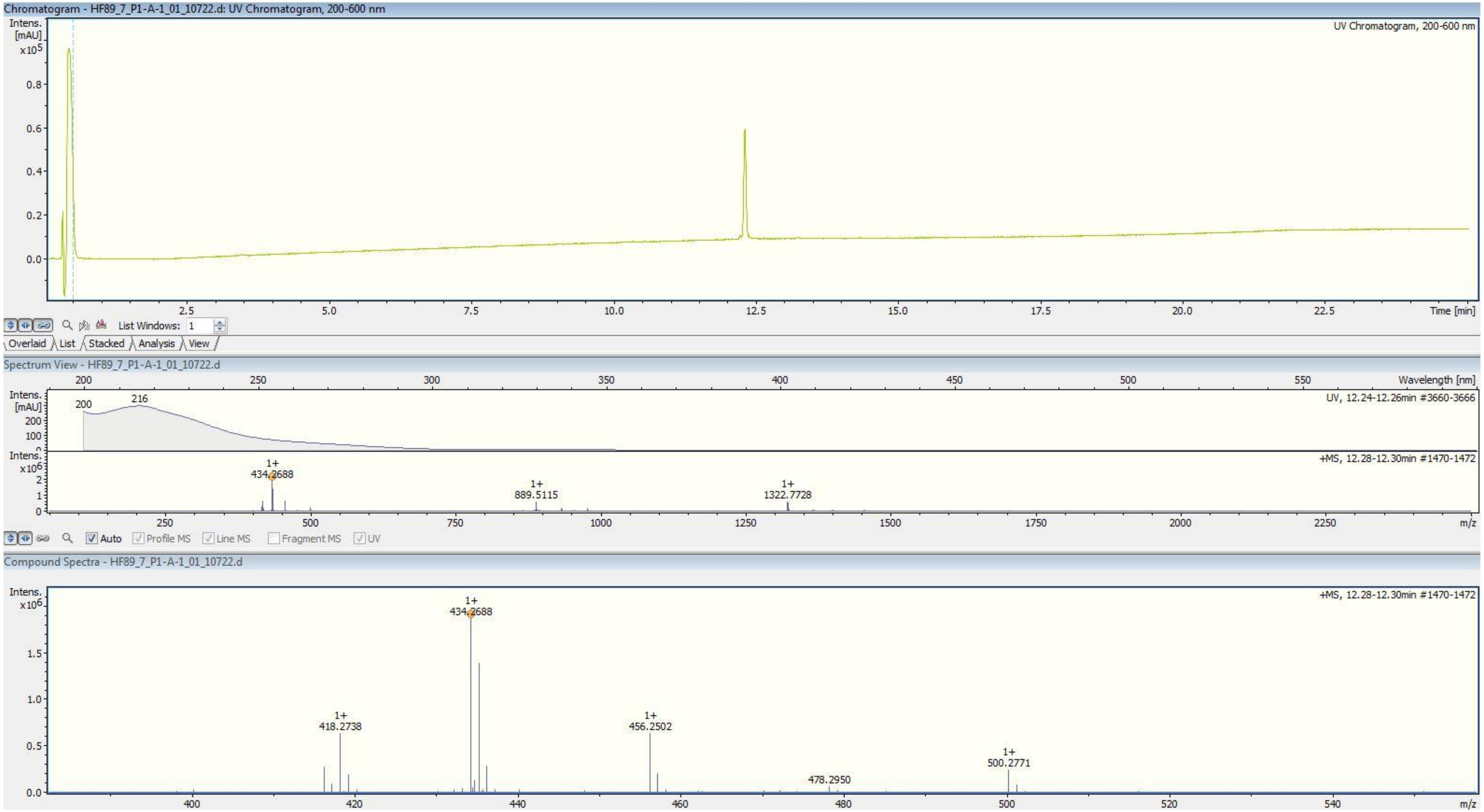

Figure S3. HPLC-HRESIMS data of **1**.

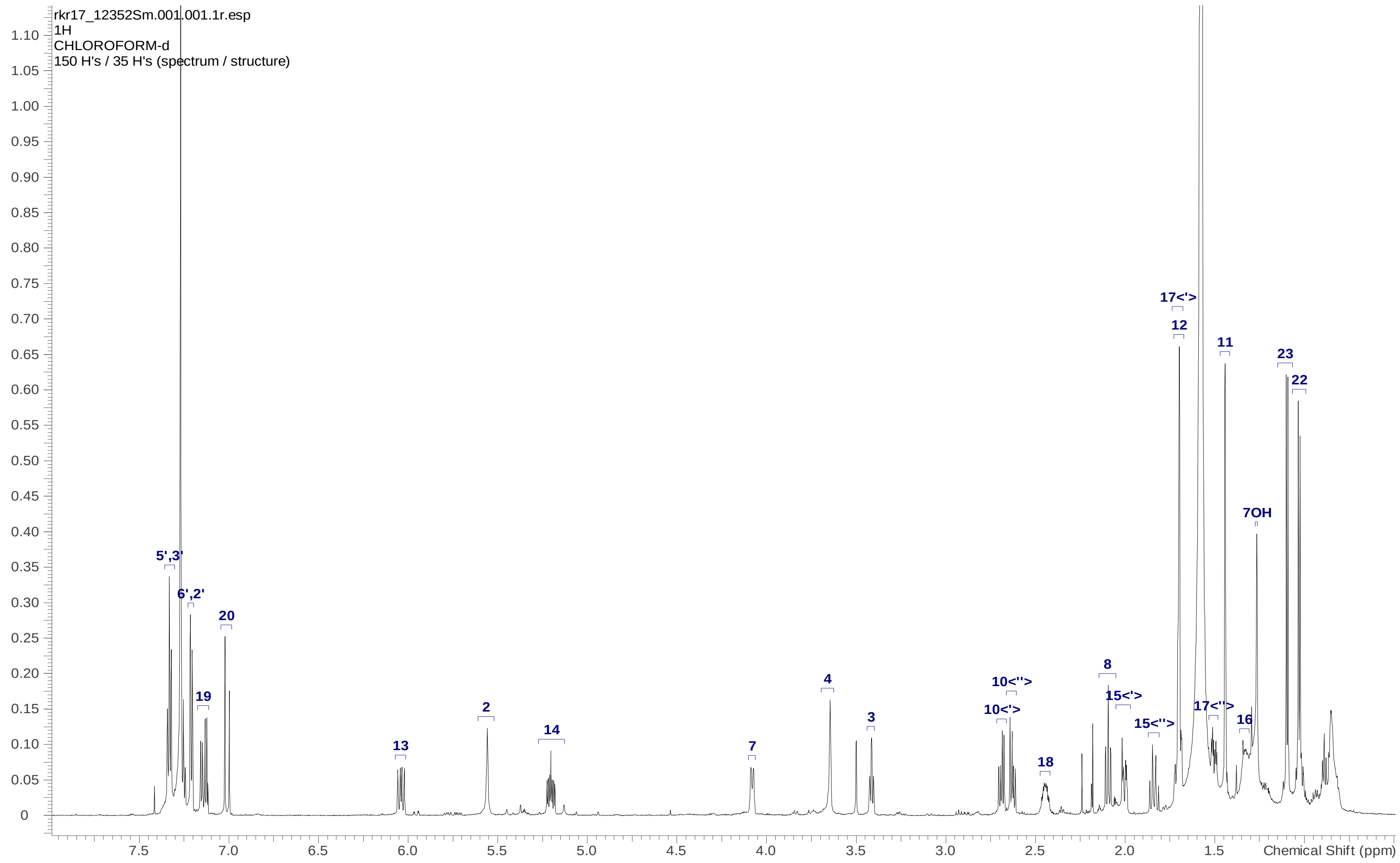

Figure S4. ^{1}H NMR spectrum (700 MHz, $CDCl_3$) of **1**.

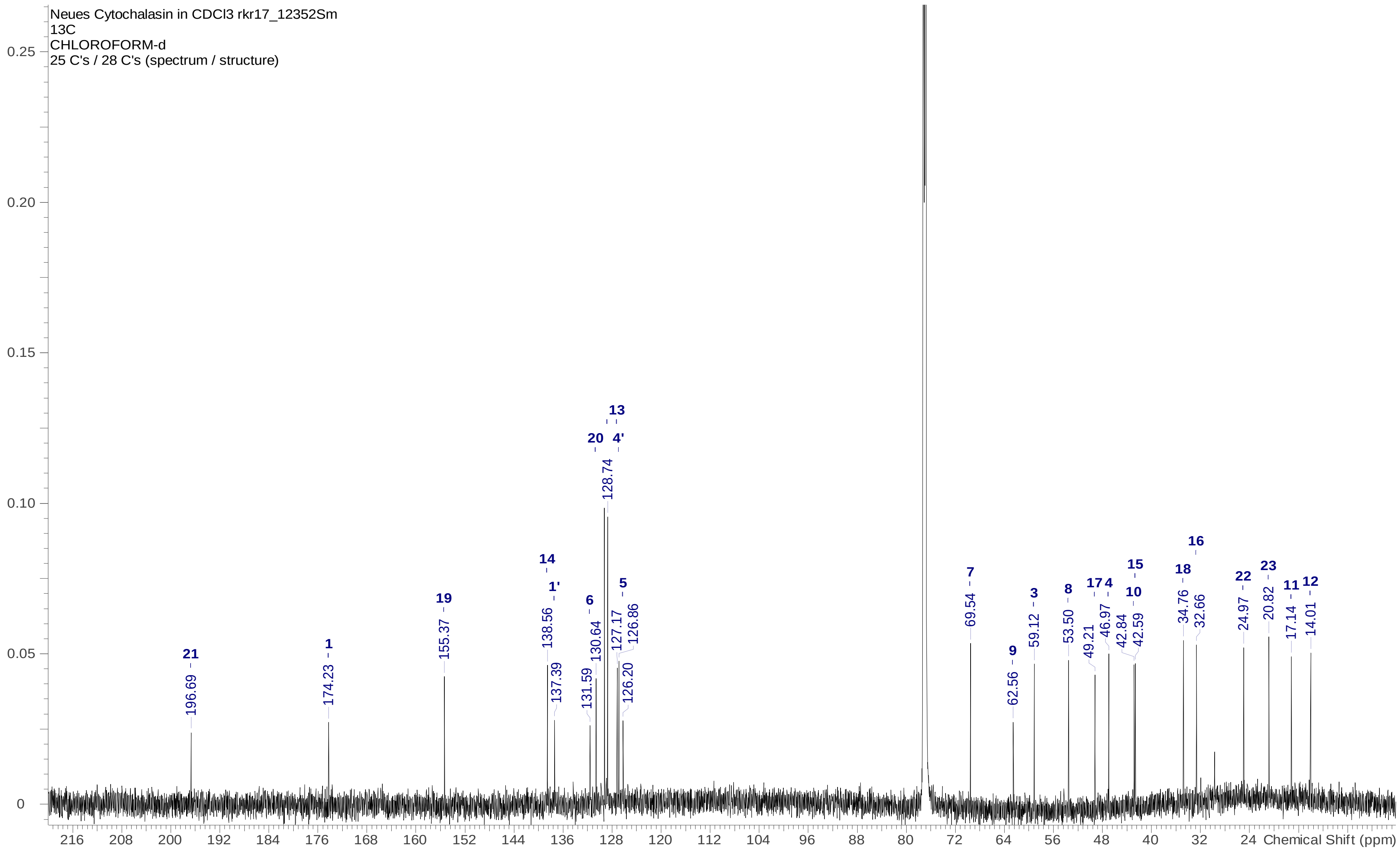

Figure S5. ^{13}C NMR spectrum (175 MHz, $CDCl_3$) of **1**.

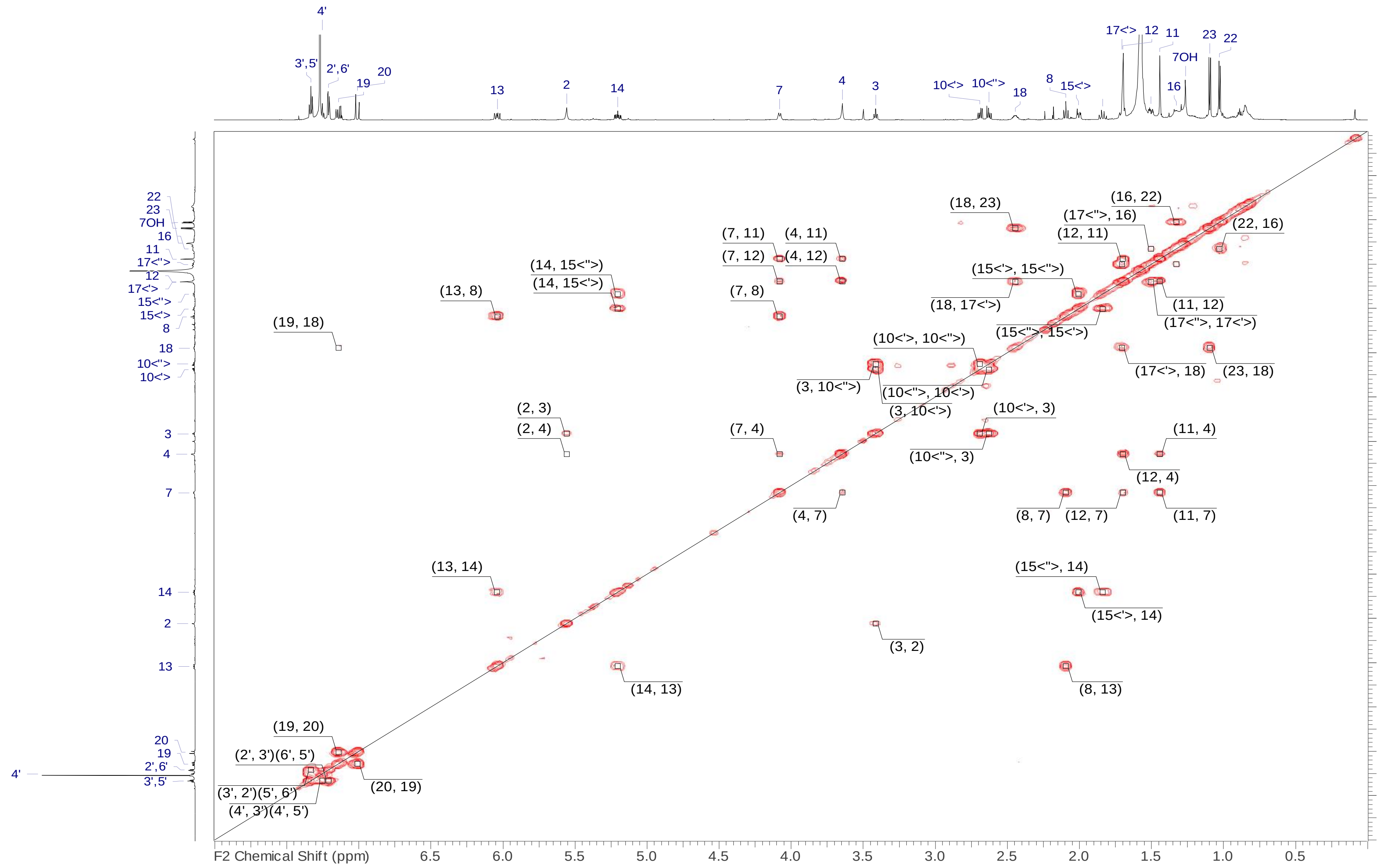

Figure S6. COSY NMR spectrum (700 MHz, $CDCl_3$) **1**.

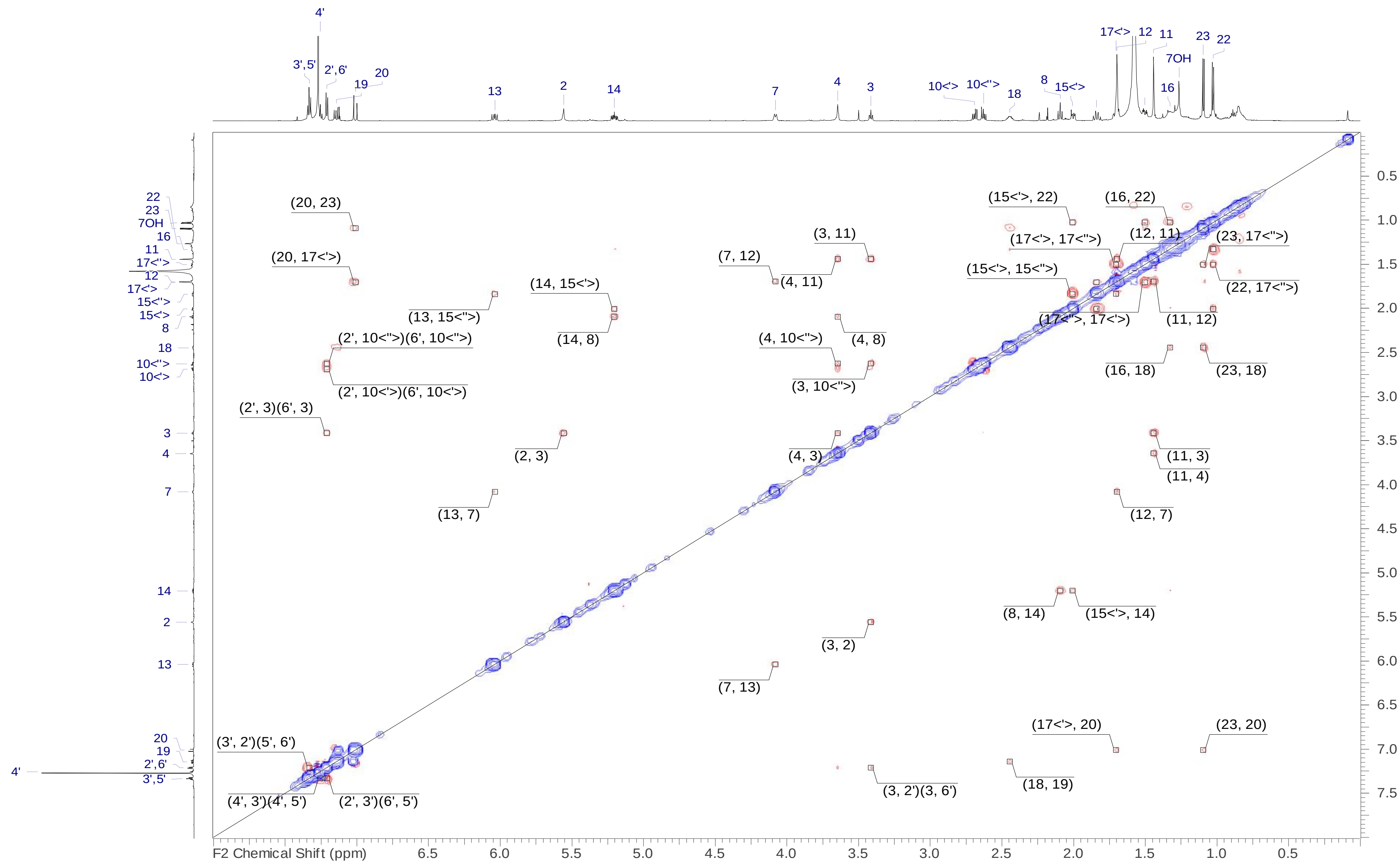

Figure S7. ROESY NMR spectrum (700 MHz, $CDCl_3$) **1**.

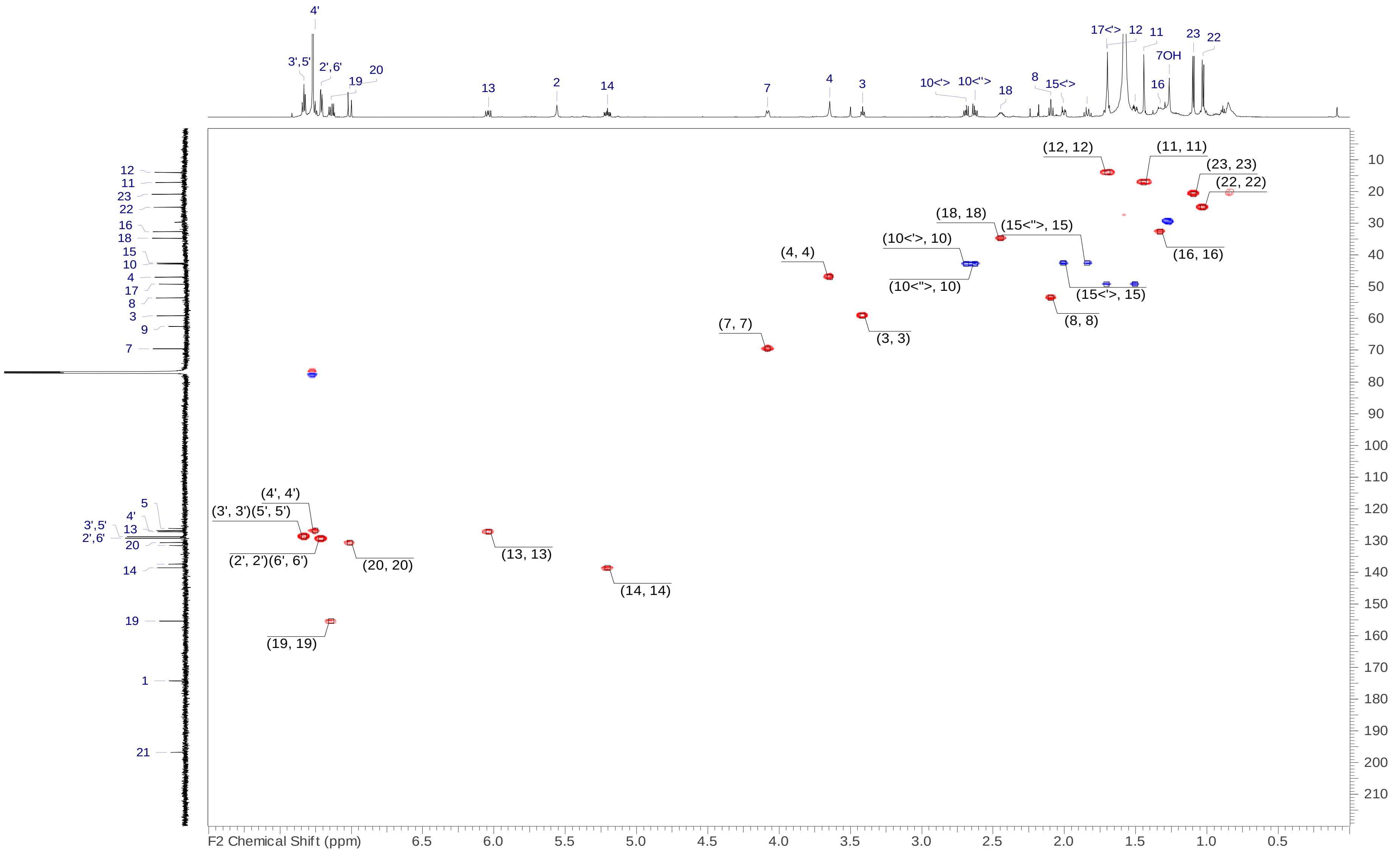

Figure S8. HSQC NMR spectrum (700 MHz, $CDCl_3$) **1**.

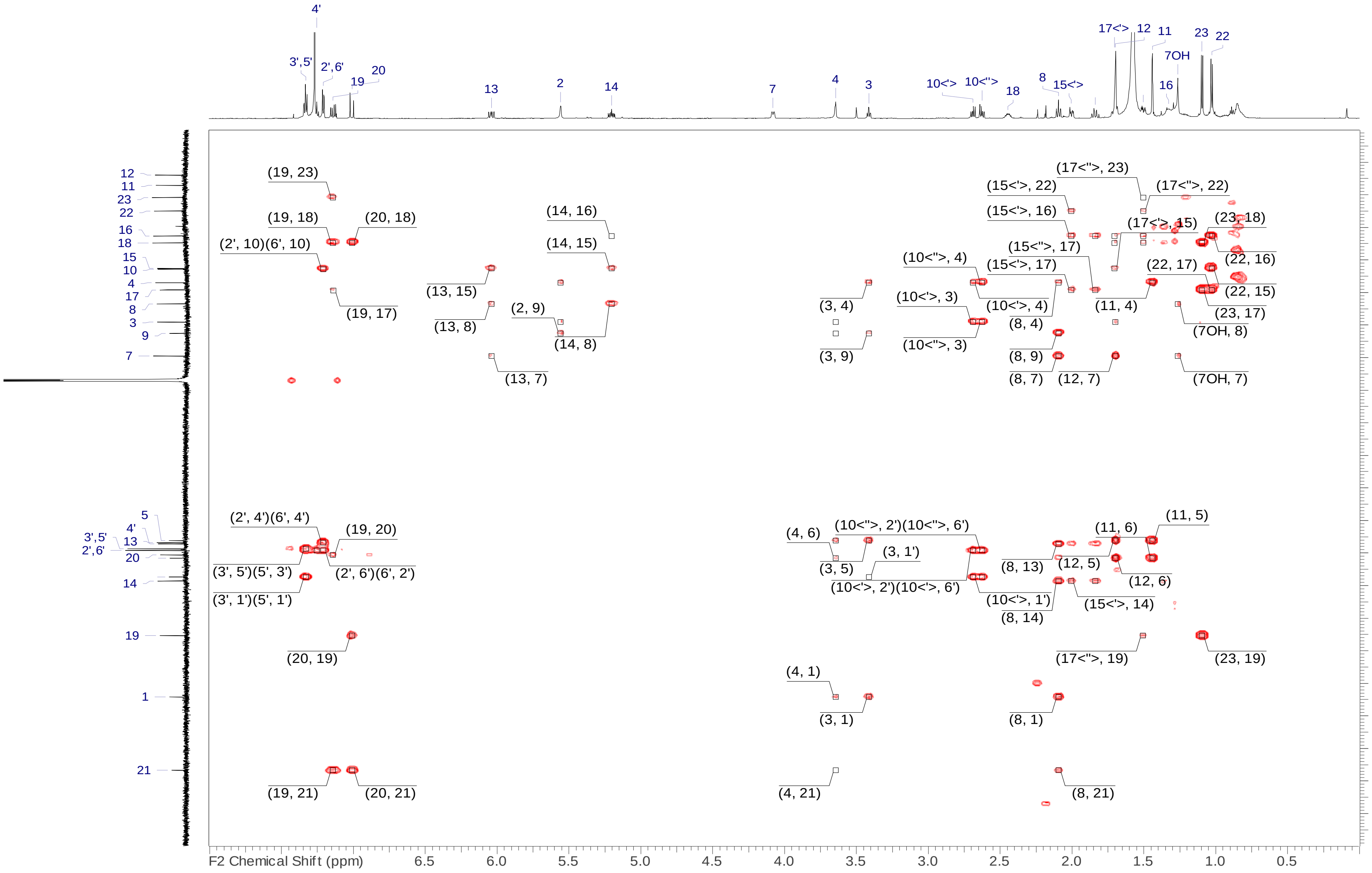

Figure S9. HMBC NMR spectrum (700 MHz, $CDCl_3$) **1**.

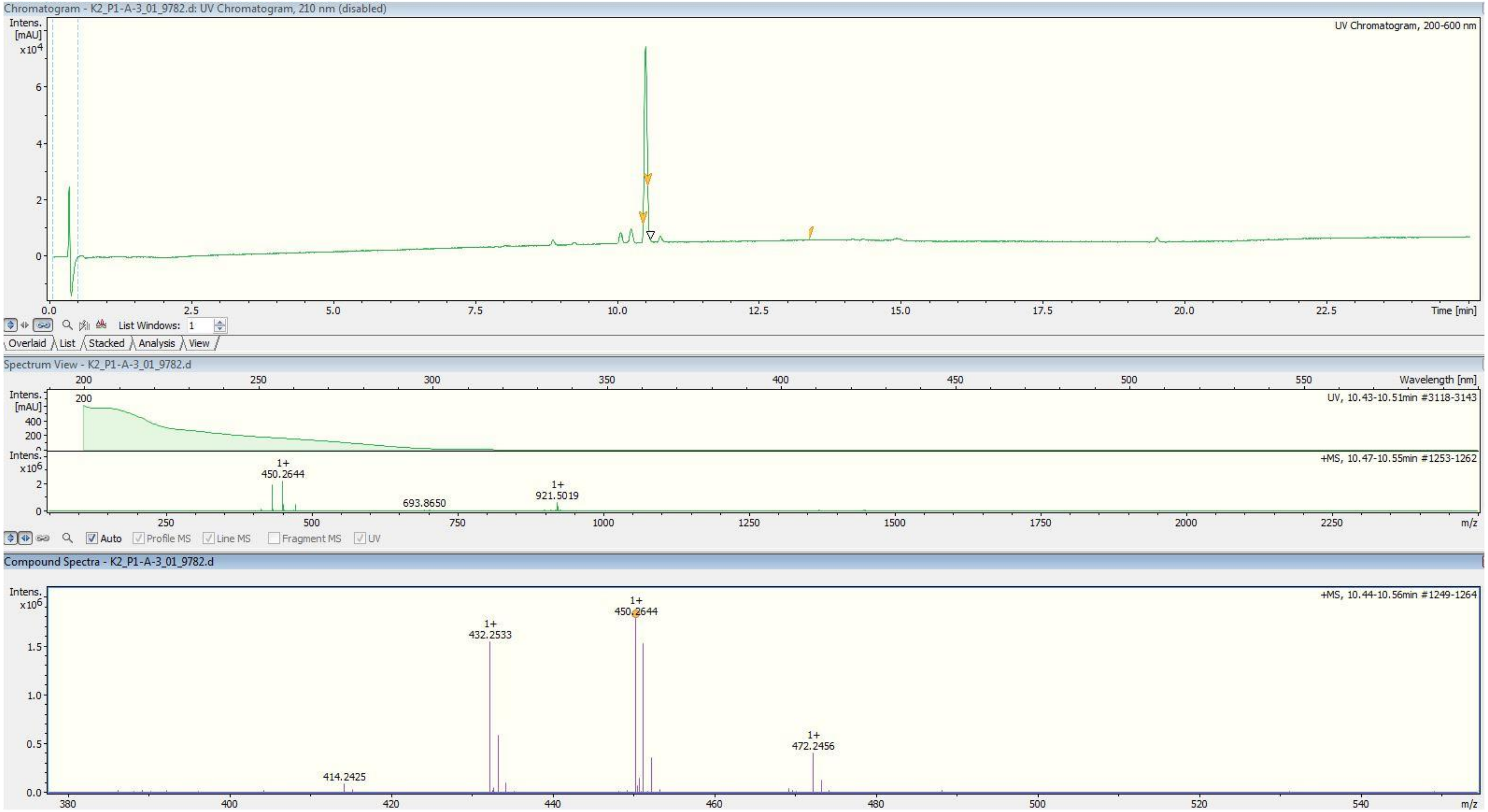

Figure S10. HPLC-HRESIMS data of of **2**.

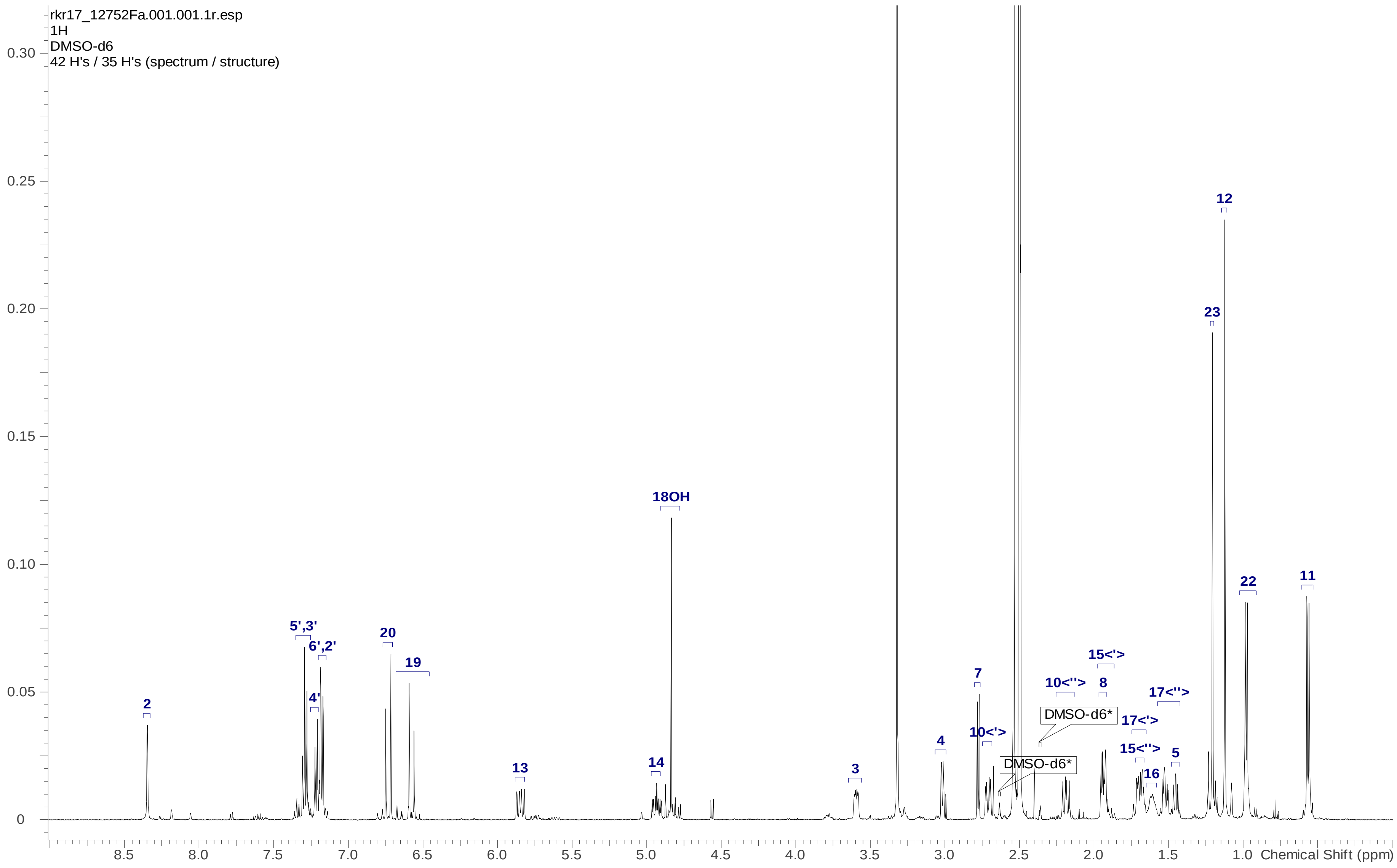

Figure S11. ^{1}H NMR spectrum (500 MHz, DMSO-d_6) of **2**.

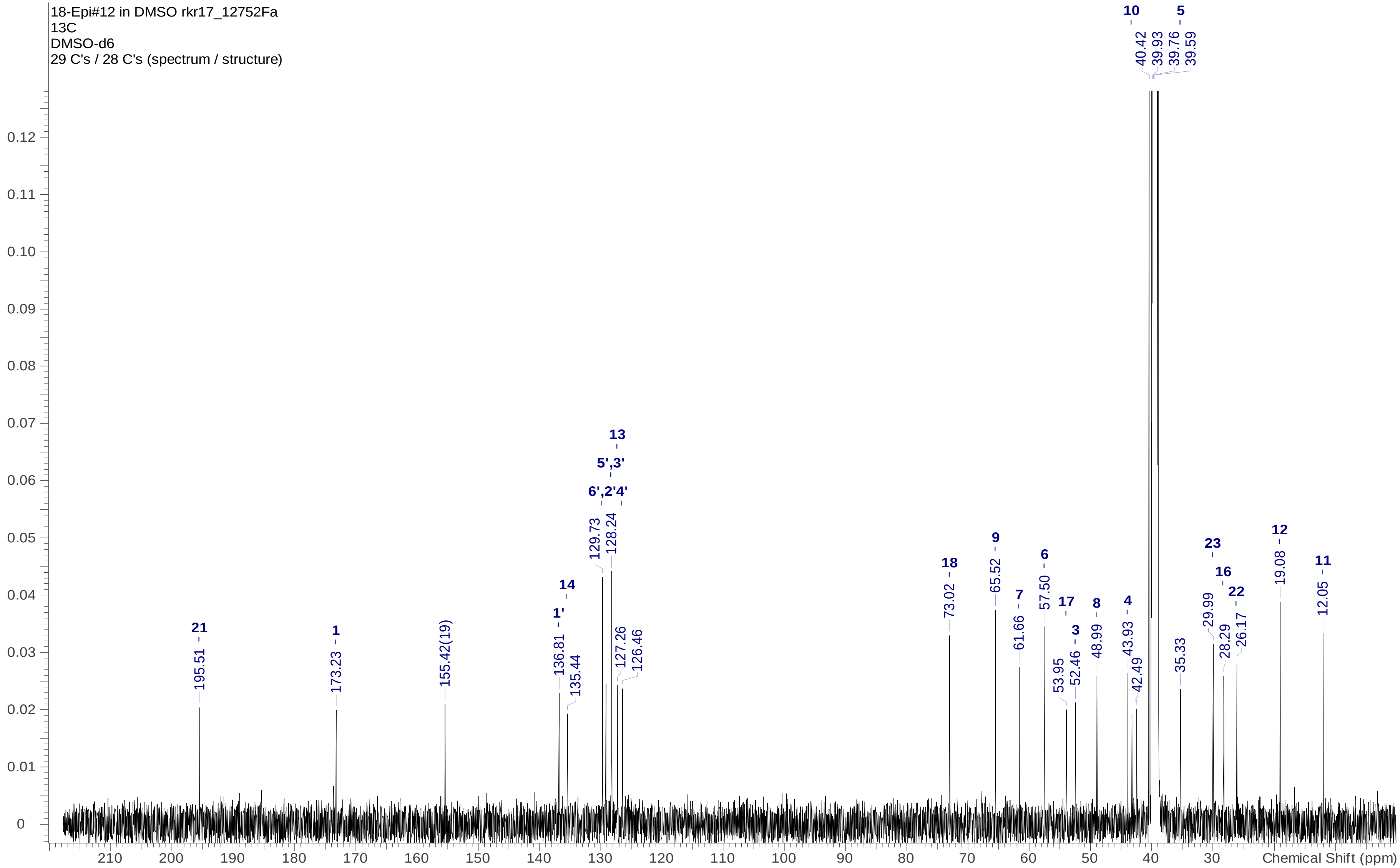

Figure S12. ^{13}C NMR spectrum (125 MHz, DMSO-d_6) of **2**.

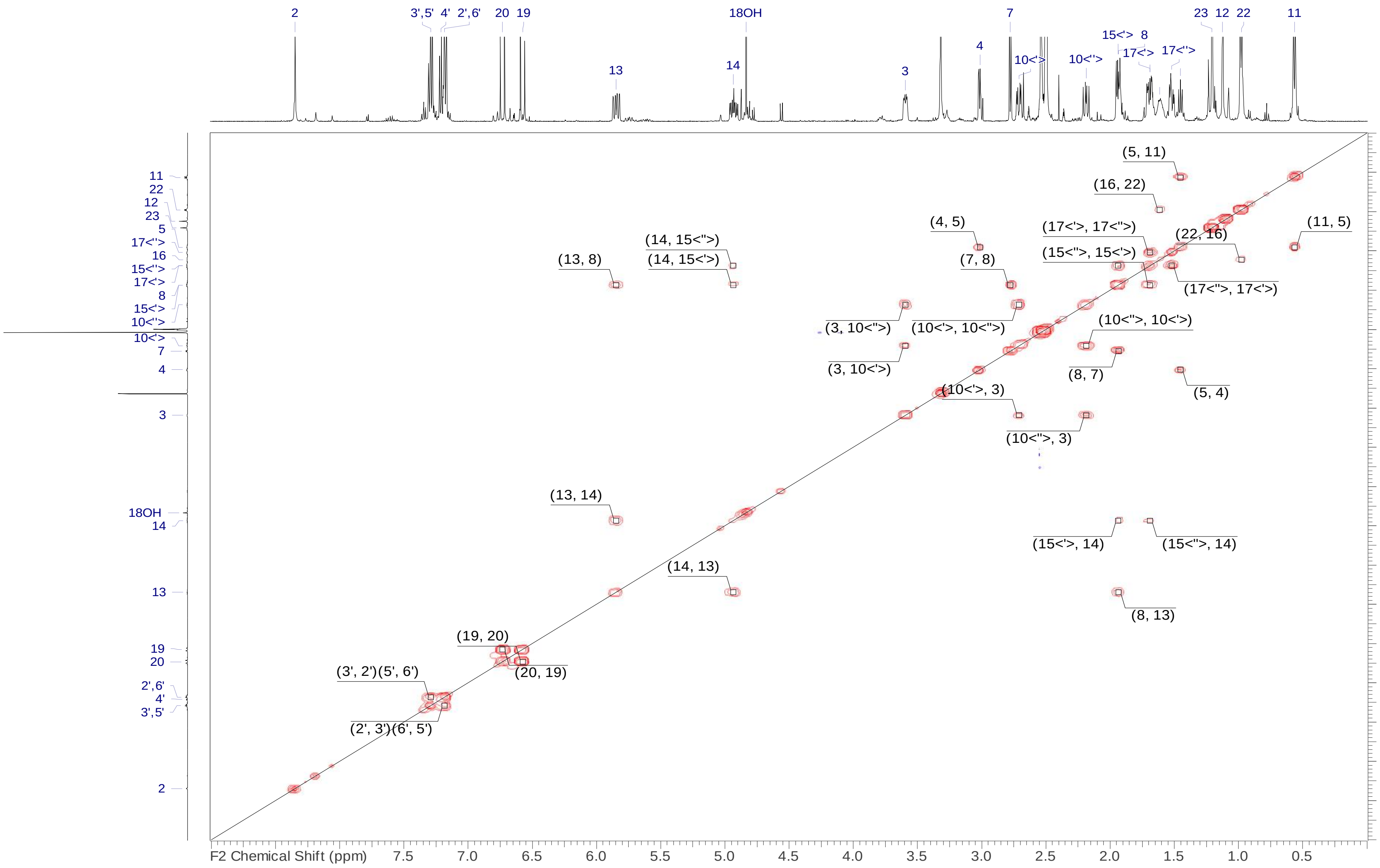

Figure S13. COSY NMR spectrum (500 MHz, DMSO-d_6) of **2**.

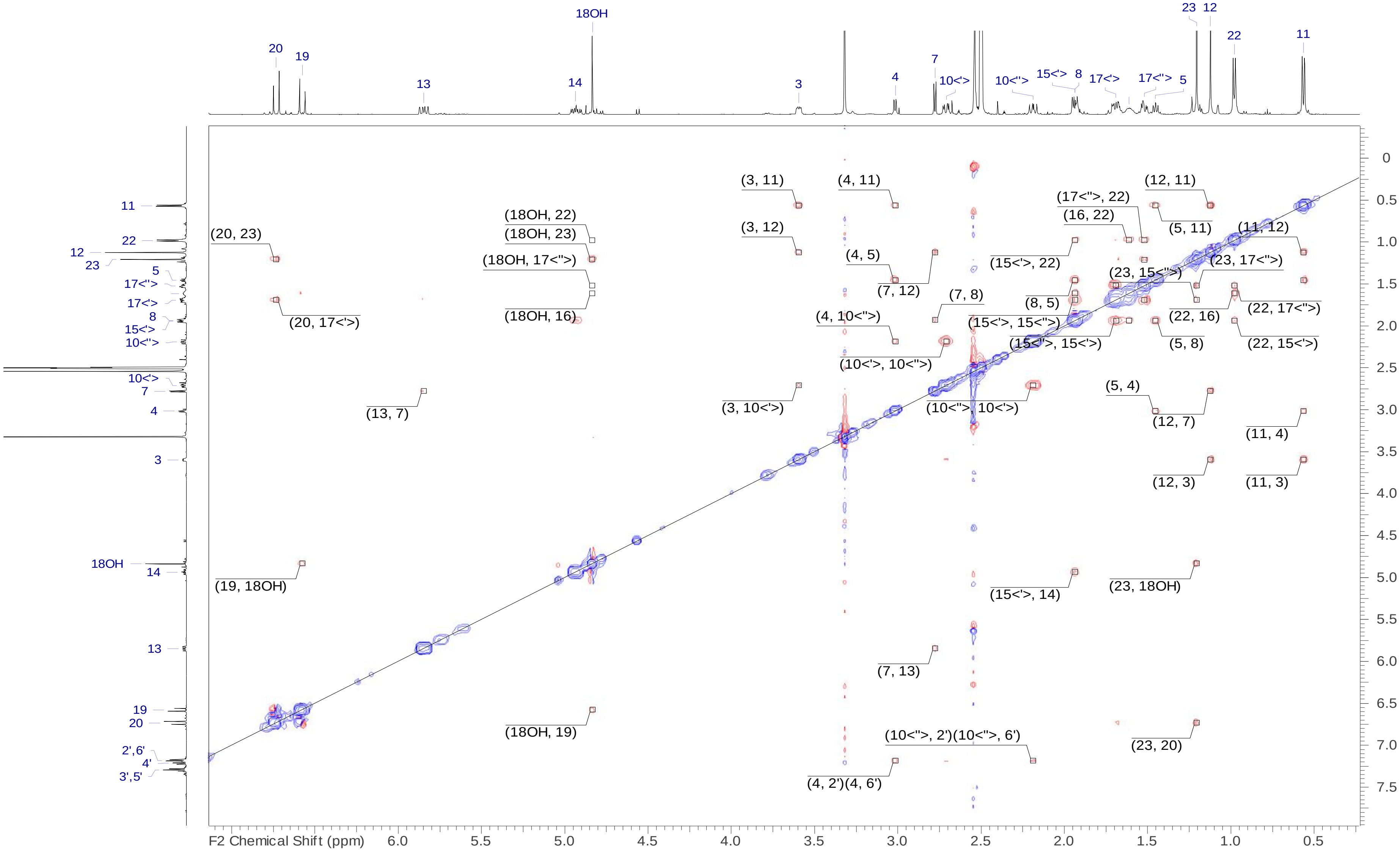

Figure S14. ROESY NMR spectrum (500 MHz, DMSO-d_6) of **2**.

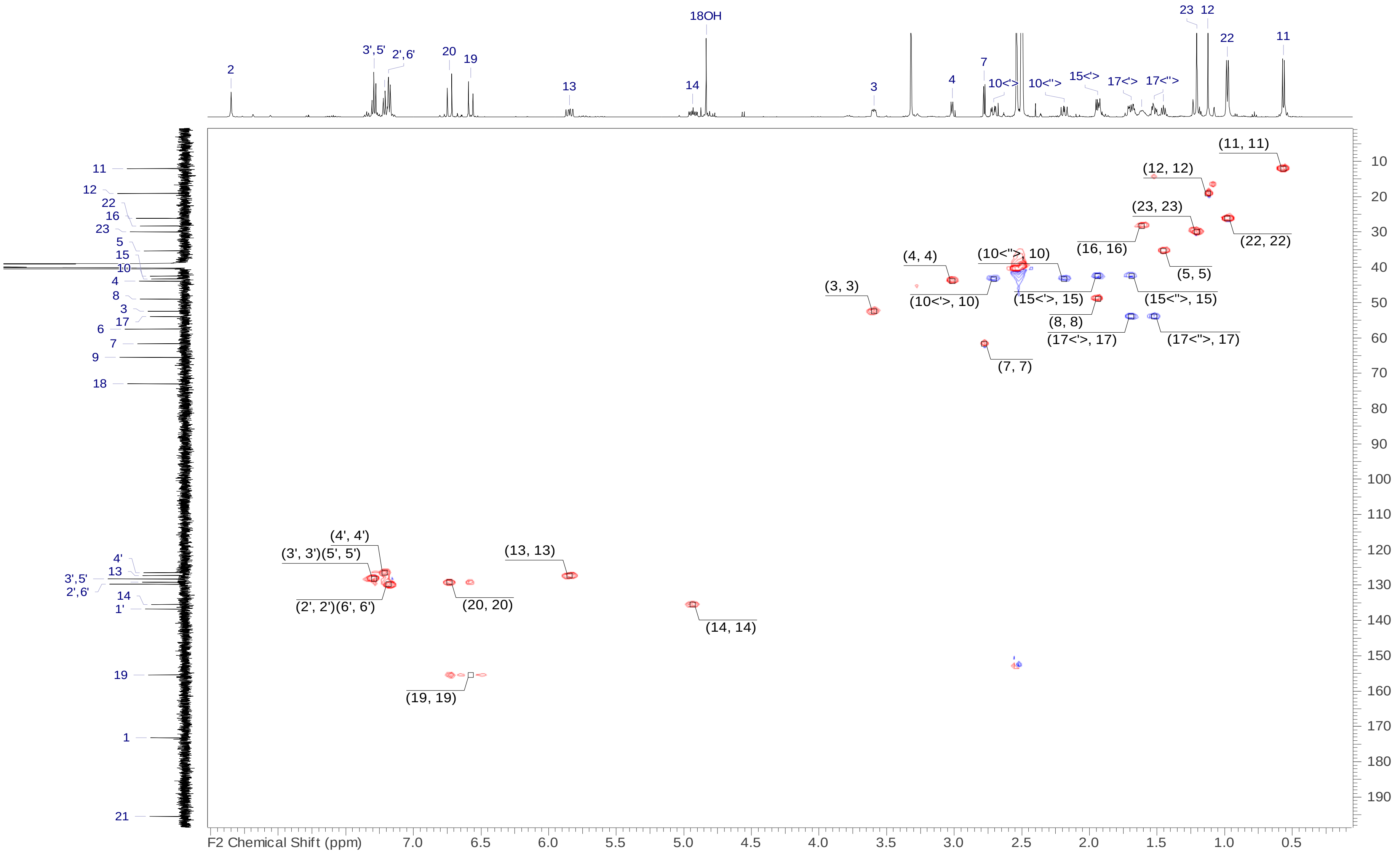

Figure S15. HSQC NMR spectrum (500 MHz, DMSO-d_6) of **2**.

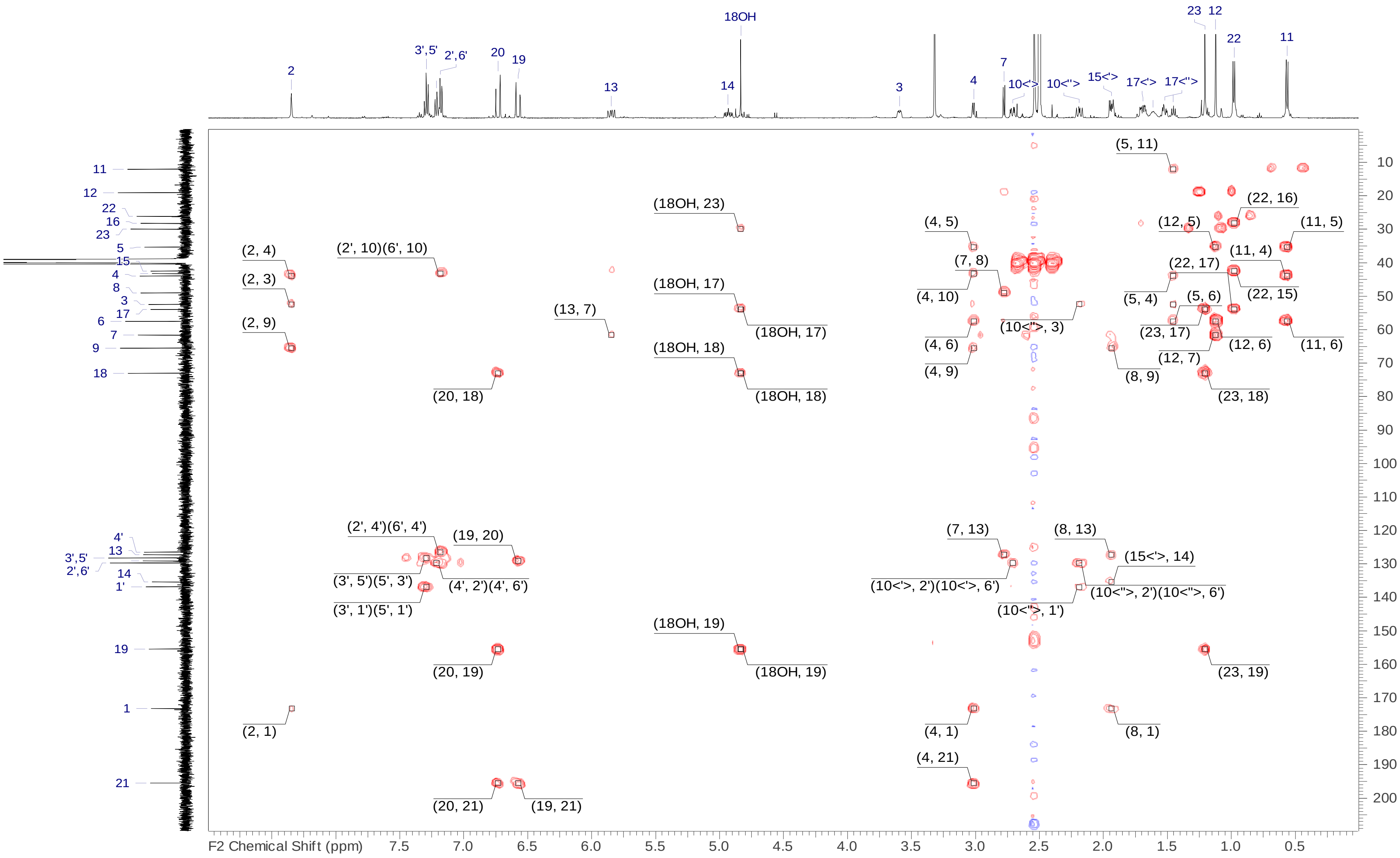

Figure S16. HMBC NMR spectrum (500 MHz, DMSO-d_6) of **2**.

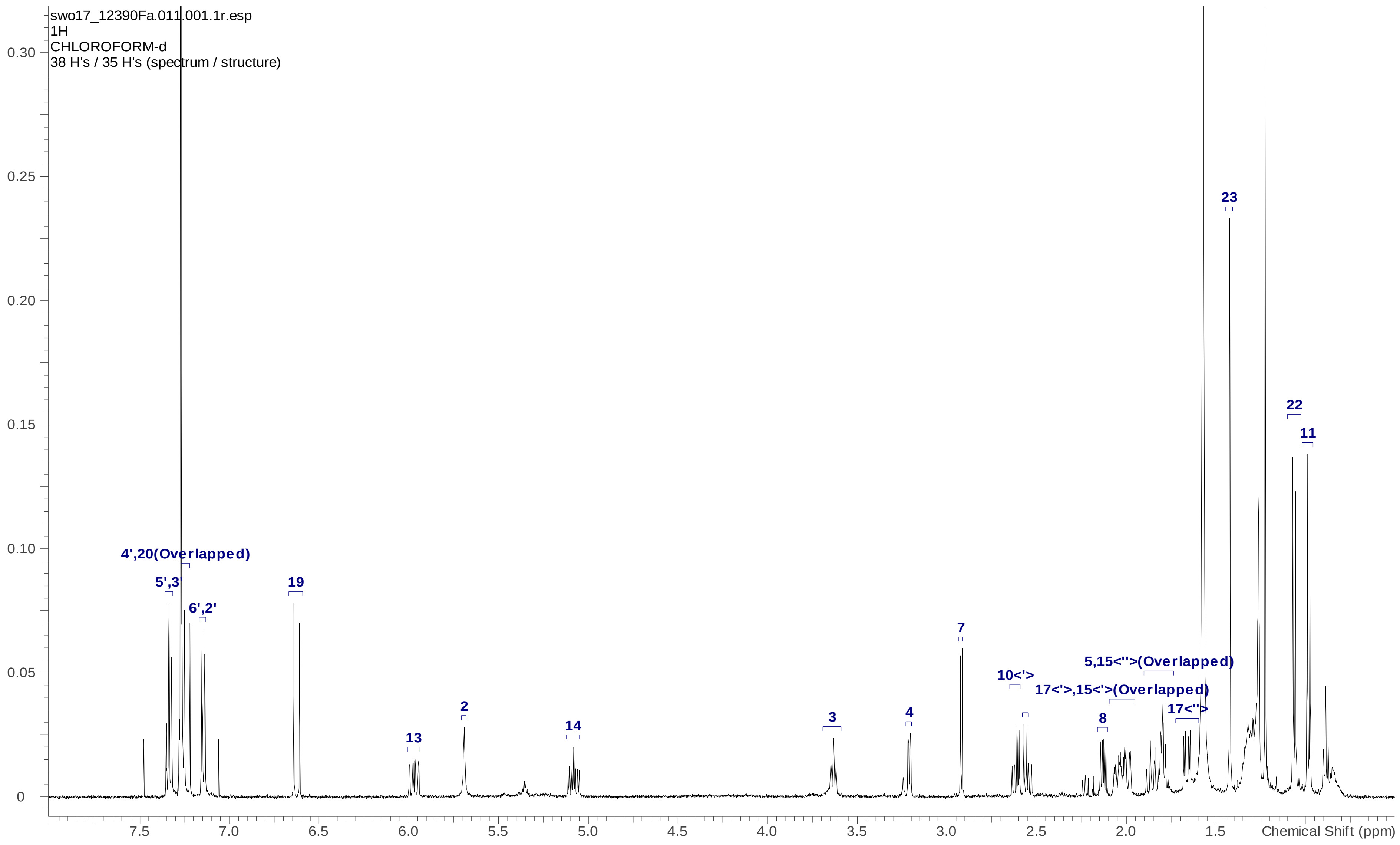

Figure S17. ^{1}H NMR spectrum (500 MHz, $CDCl_3$) of **17**.

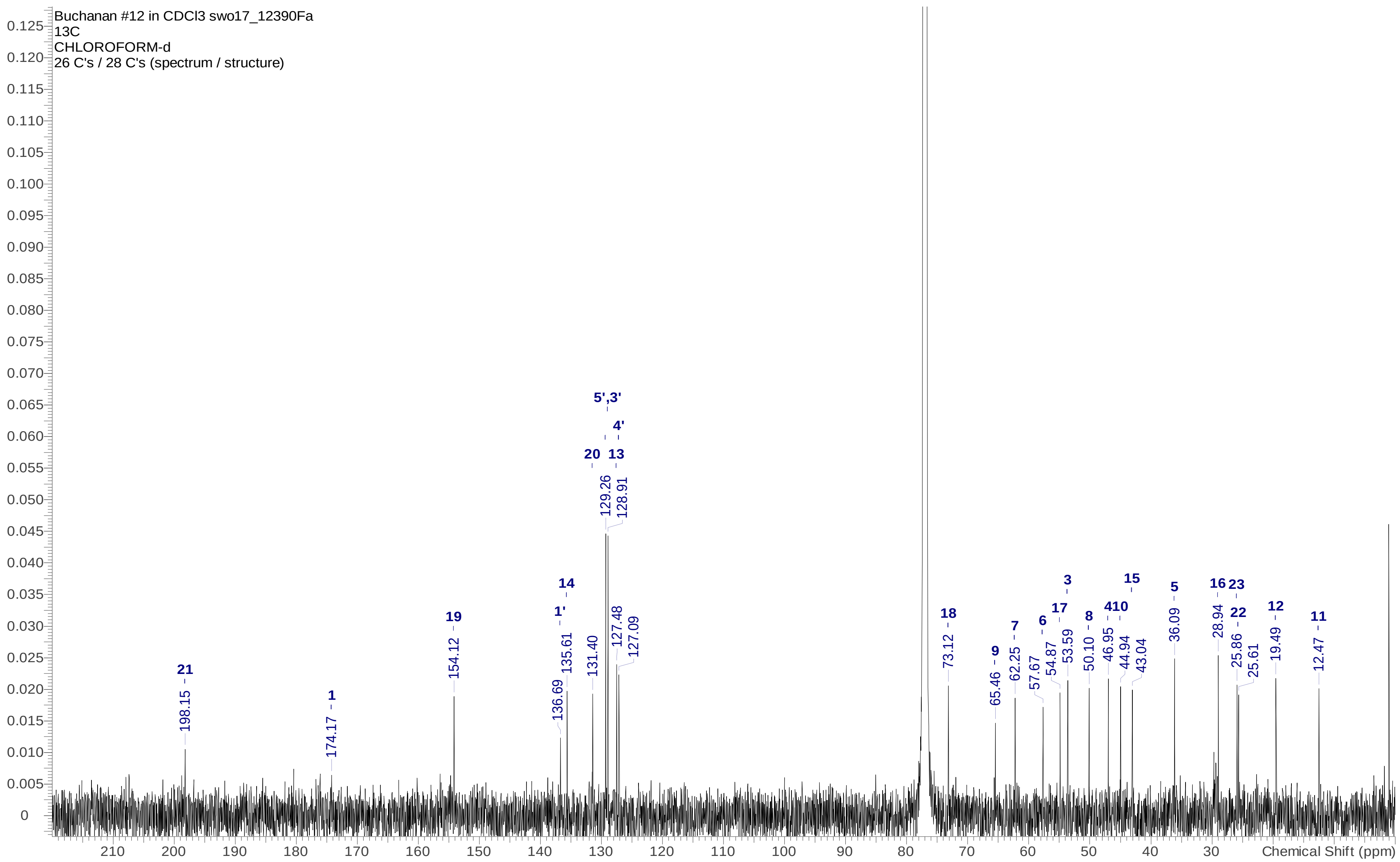

Figure S18. ^{13}C NMR spectrum (125 MHz, $CDCl_3$) of **17**.

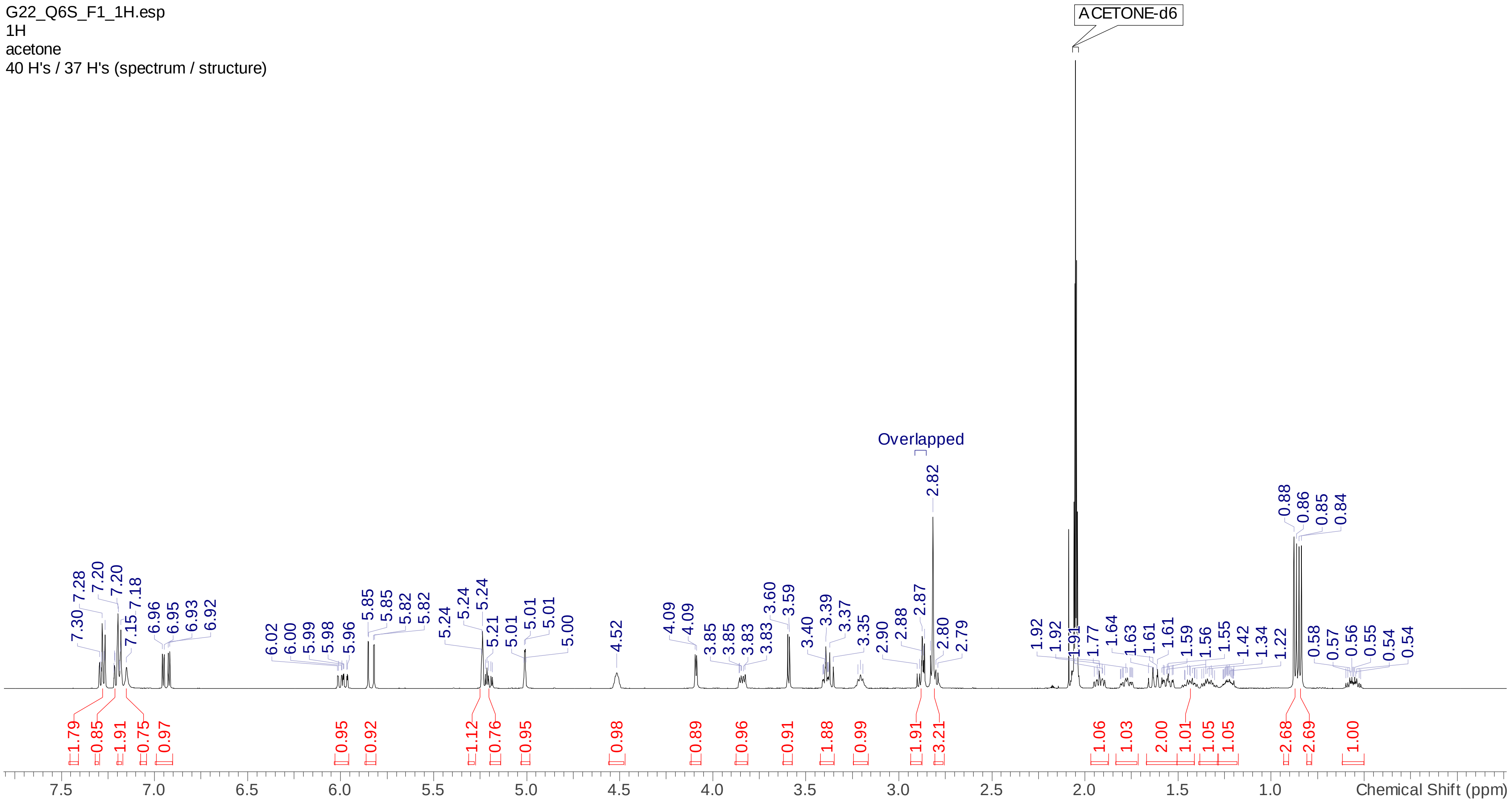

Figure S19. ^{1}H NMR spectrum (500 MHz, Acetone-d6) of cytochalasin B (**4**)

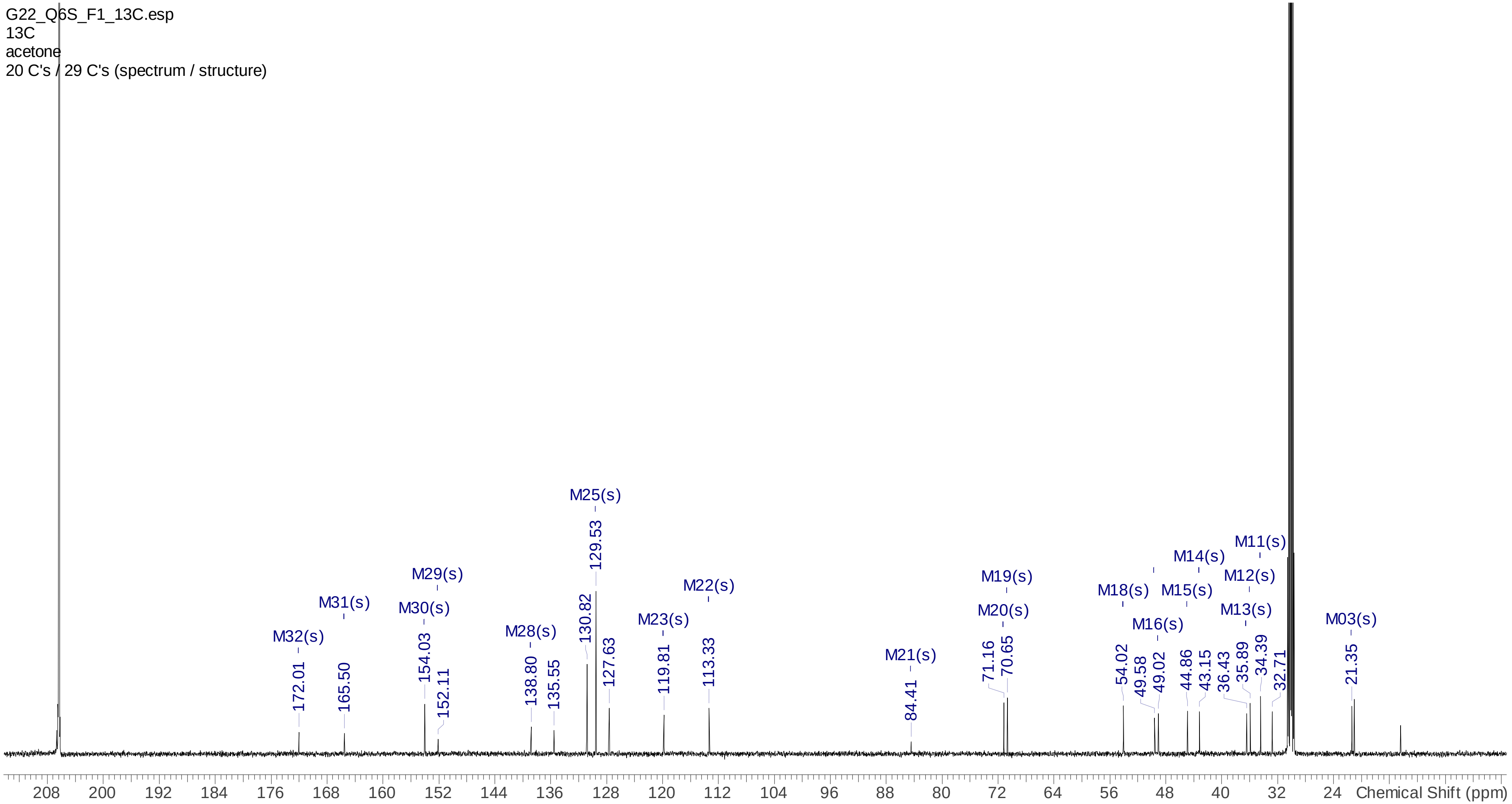

Figure S20. ^{13}C NMR spectrum (500 MHz, Acetone-d6) of cytochalasin B (**4**).

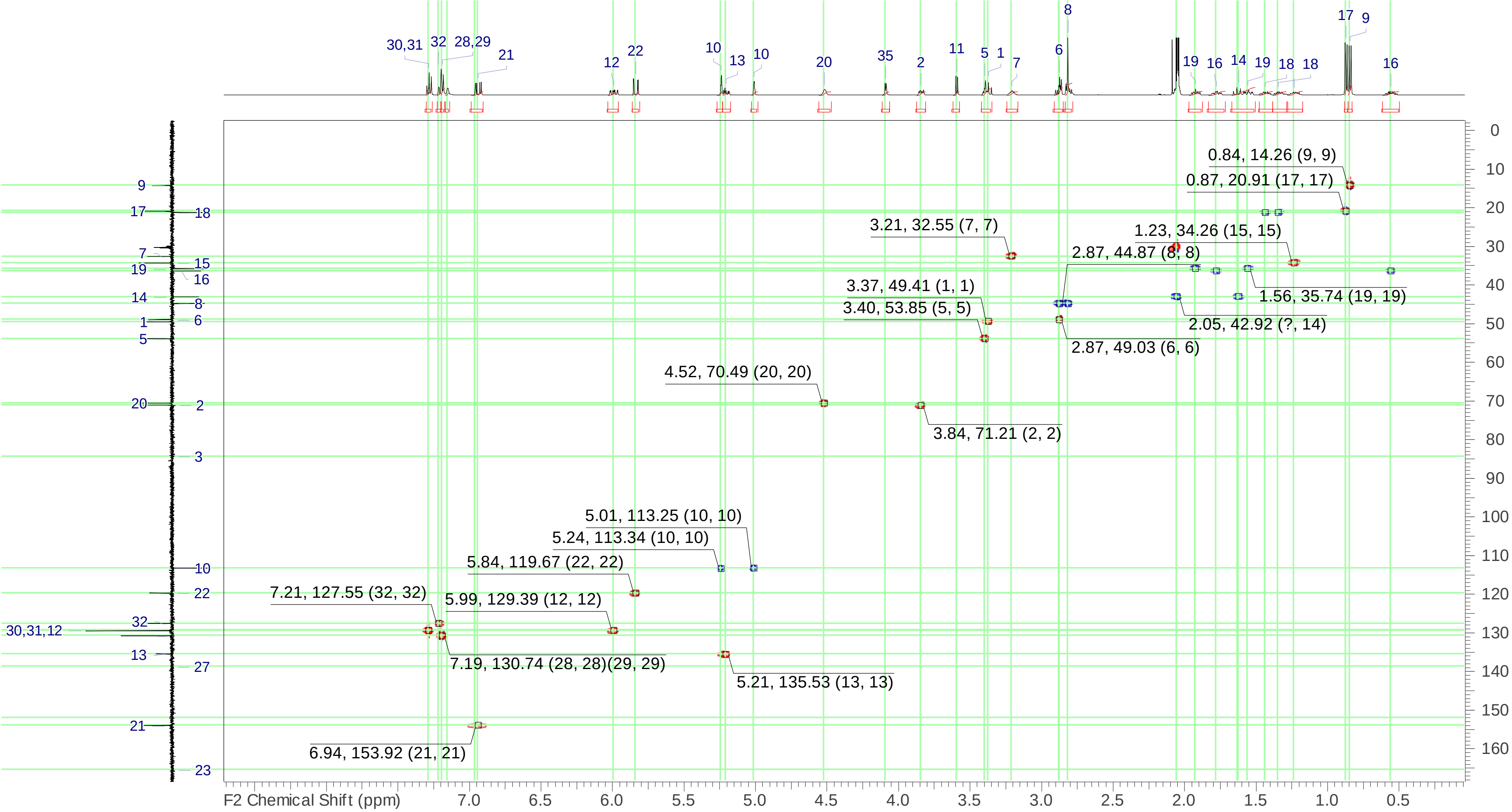

Figure S21. HSQC NMR spectrum (500 MHz, Acetone-d6) of cytochalasin B (**4**).

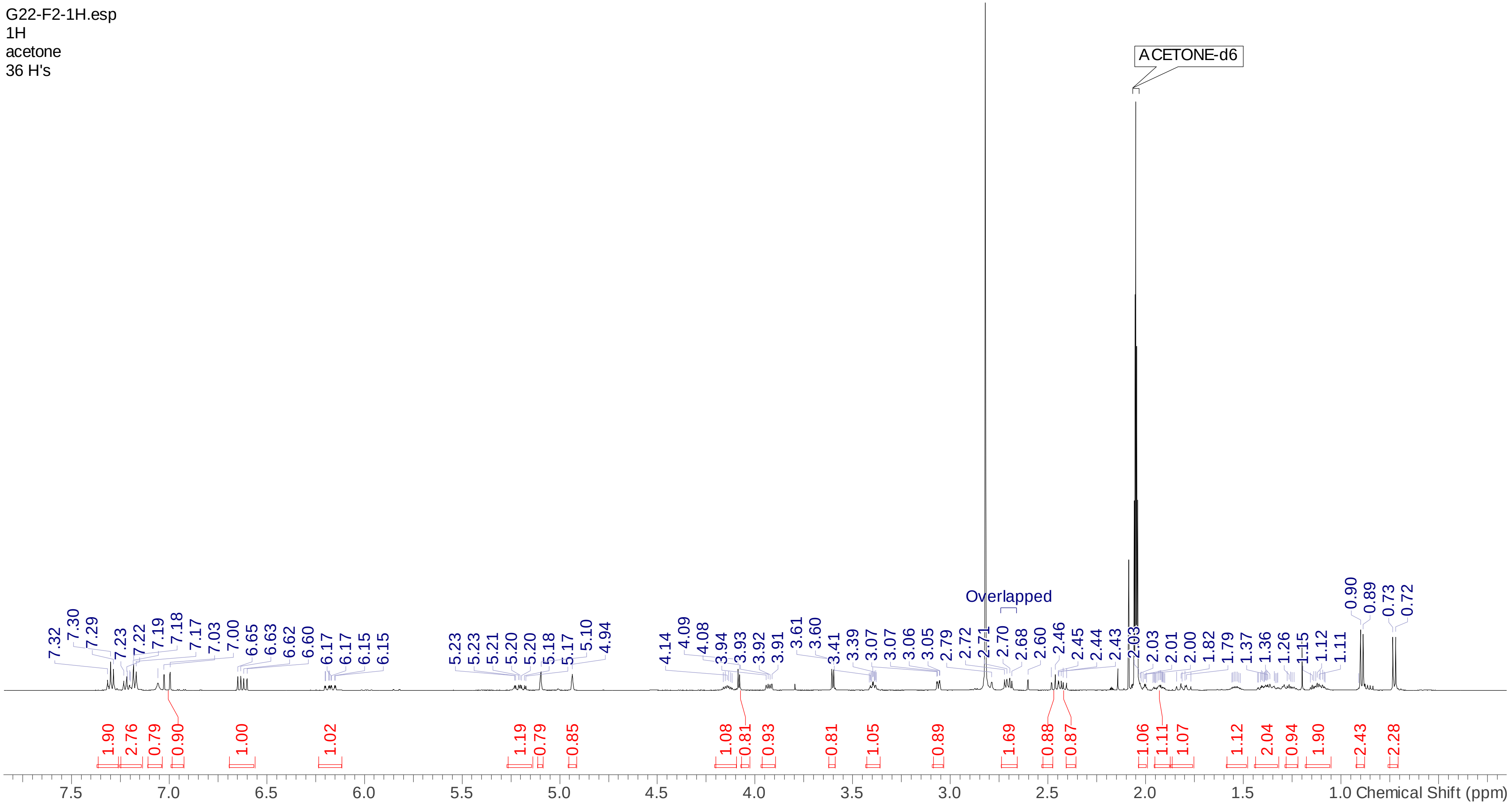

Figure S22. ^{1}H NMR spectrum (500 MHz, Acetone-d6) of deoxaphomin (**5**).

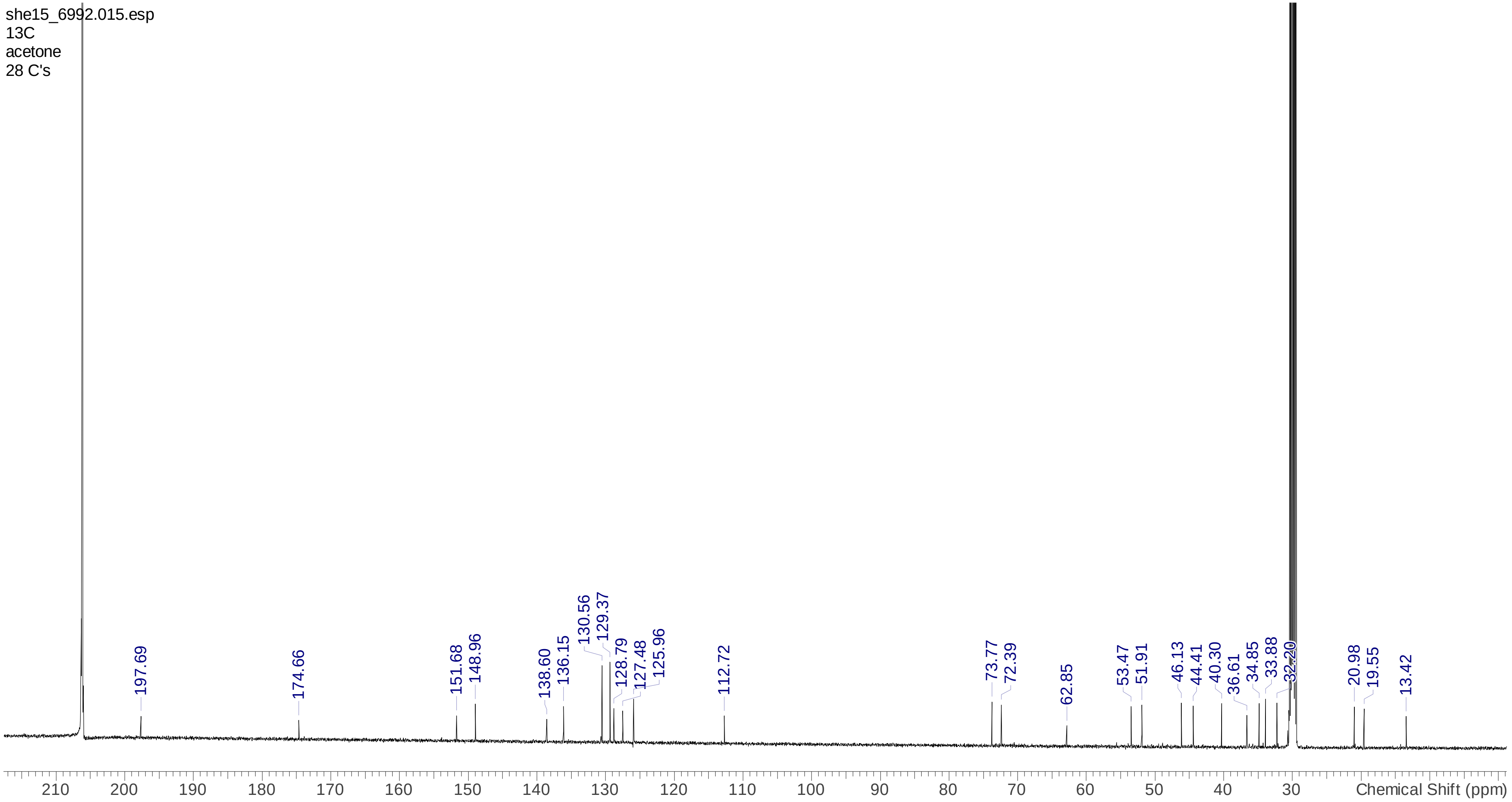

Figure S23. ^{13}C NMR spectrum (500 MHz, Acetone-d6) of deoxaphomin (**5**).

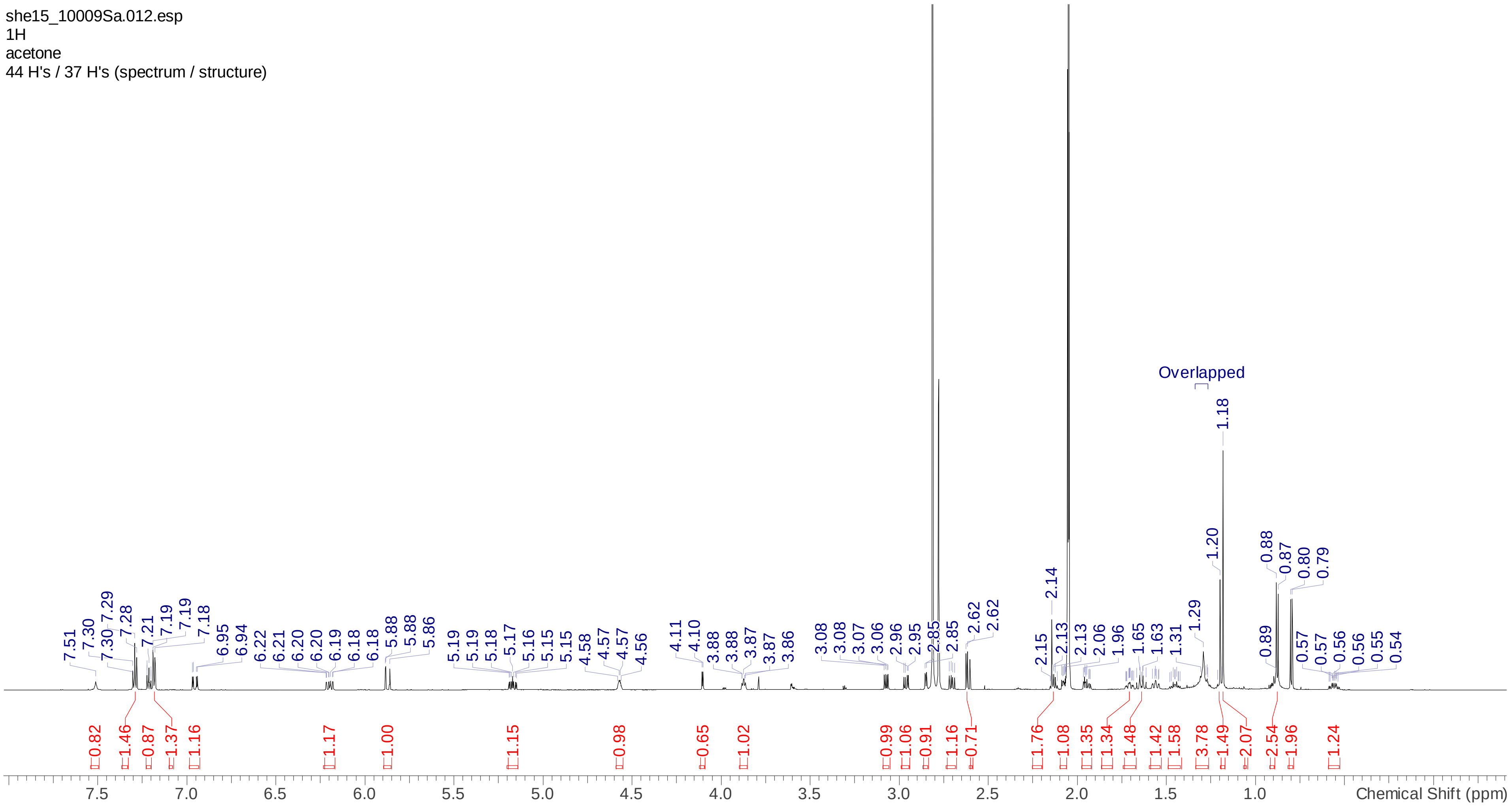

Figure S24. ^{1}H NMR spectrum (500 MHz, Acetone-d6) of cytochalasin F (**7**).

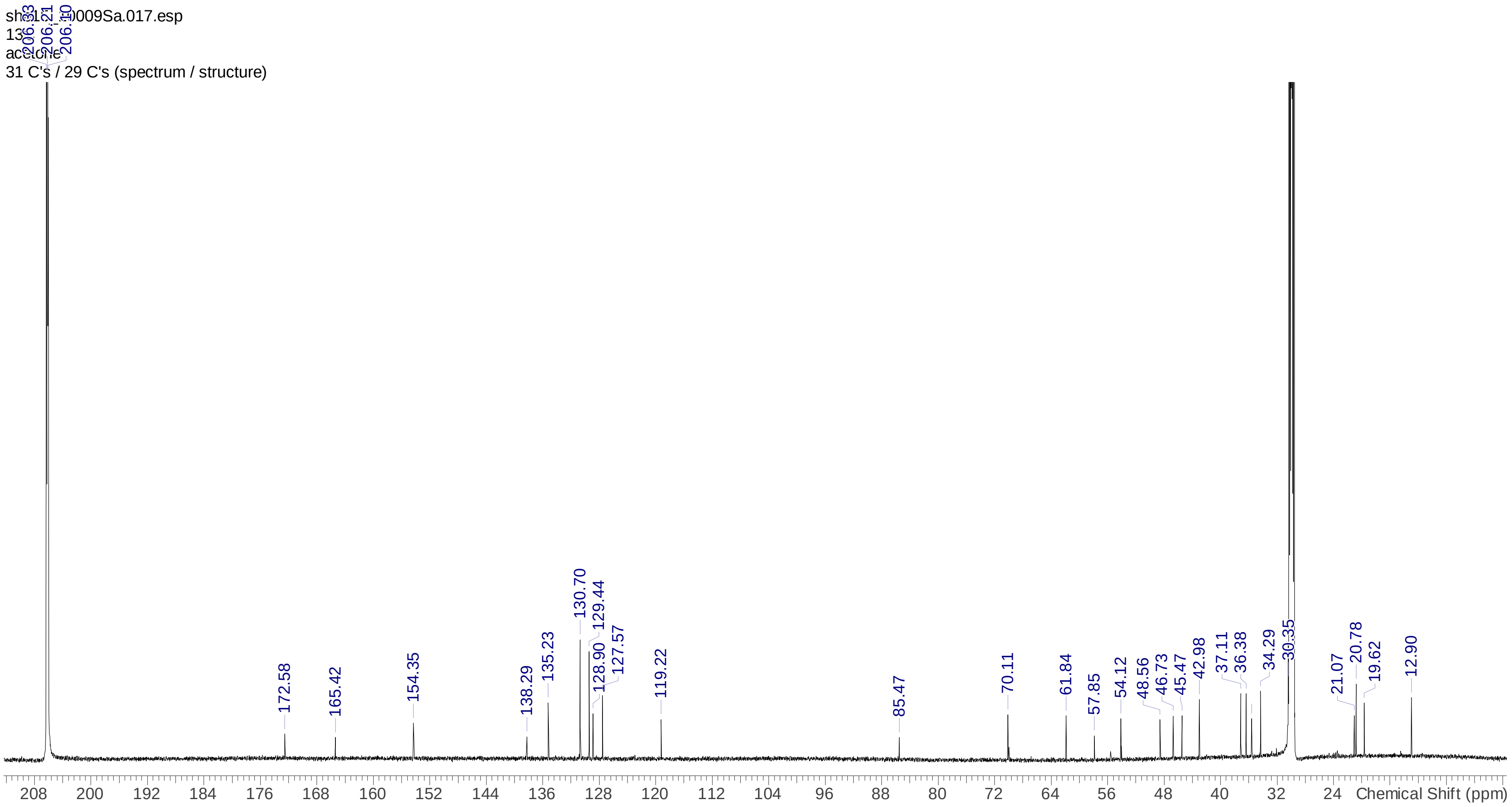

Figure S25. ^{13}C NMR spectrum (500 MHz, Acetone-d6) of cytochalasin F (**7**).

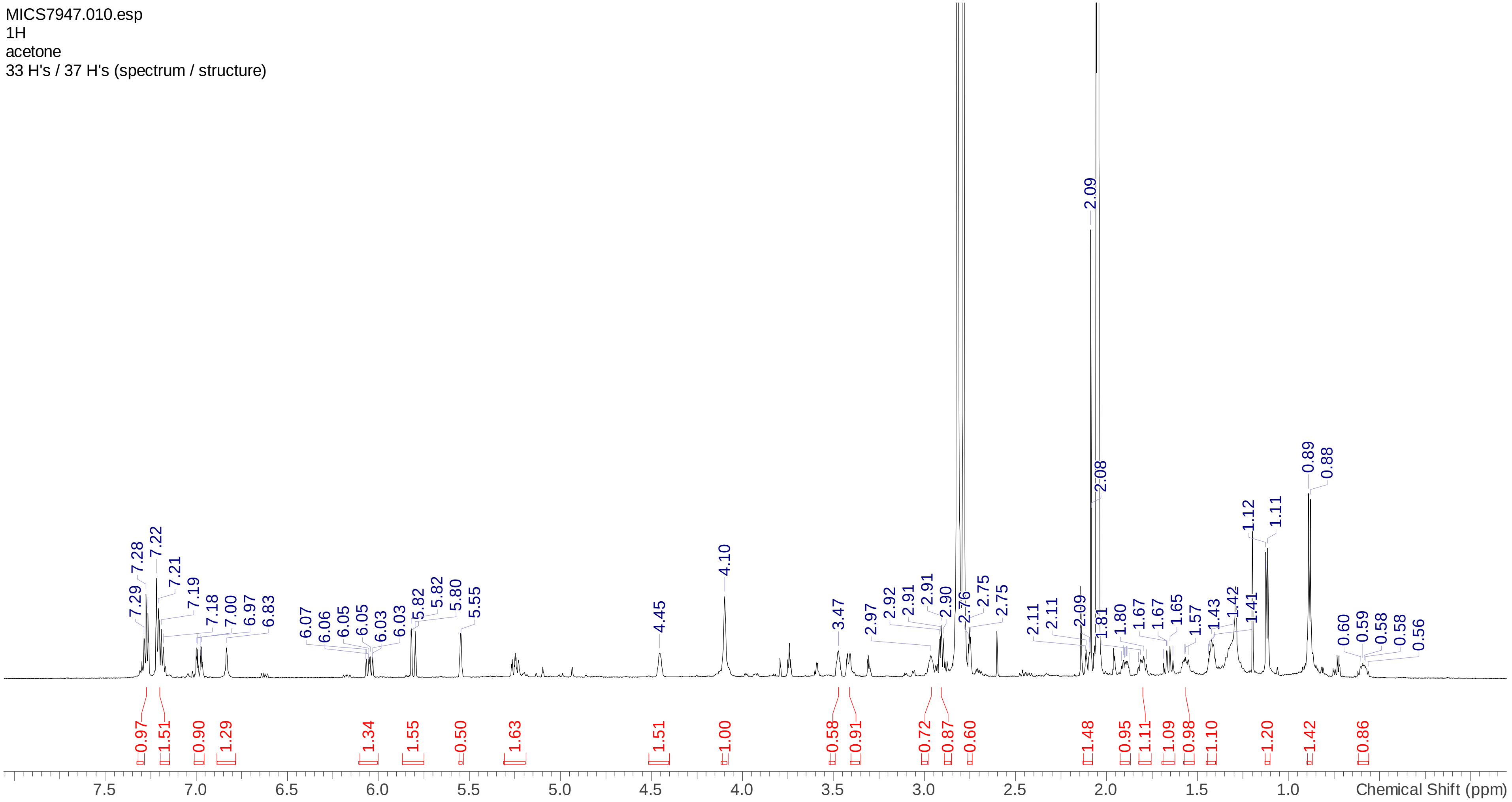

Figure S26. ^{1}H NMR spectrum (500 MHz, Acetone-d6) of cytochalasin Z2 (**11**).

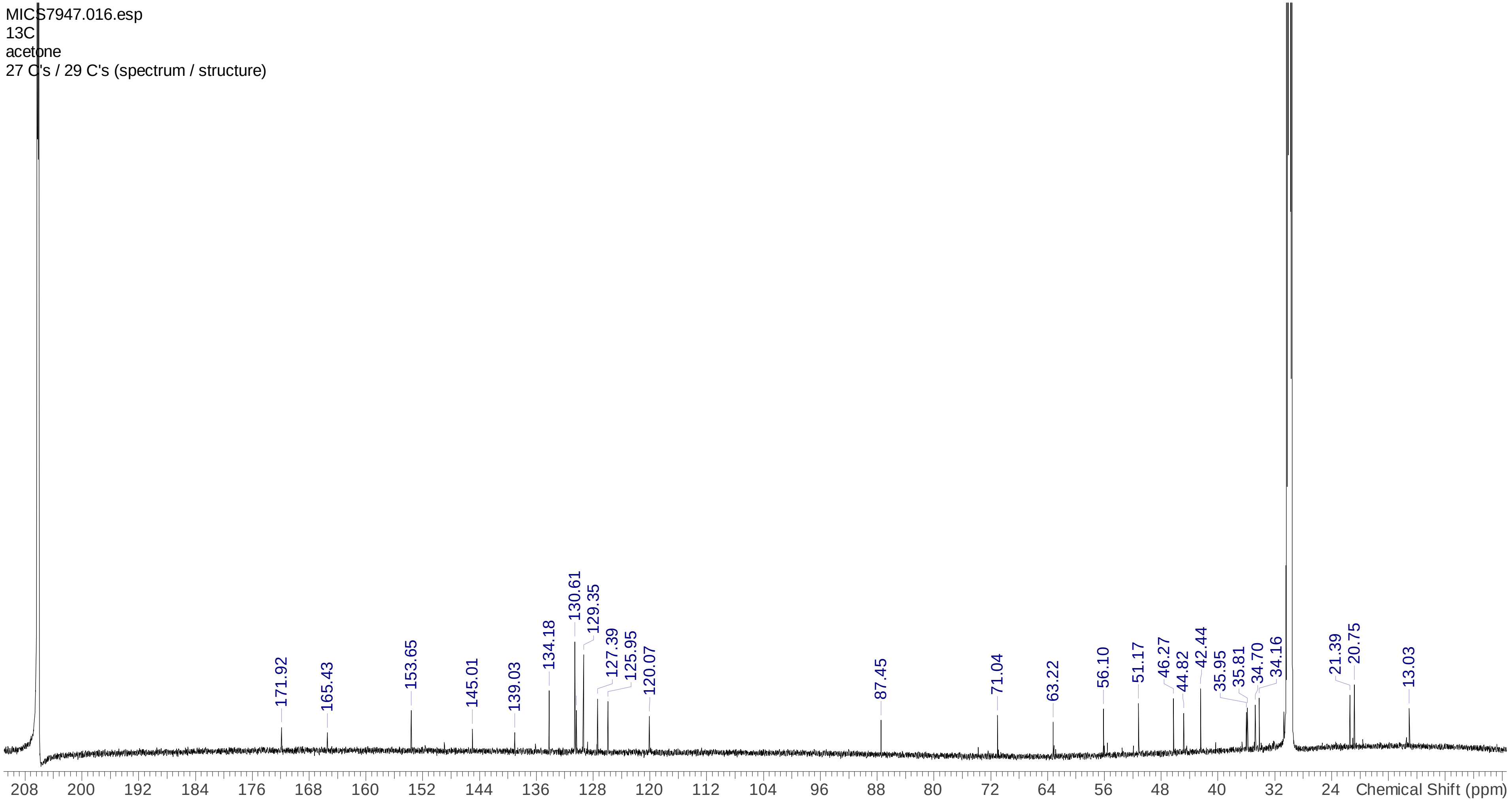

Figure S27. ^{13}C NMR spectrum (500 MHz, Acetone-d6) of cytochalasin Z2 (**11**).

Table S1. Cytochalasan treatment of U2OS cells

	compound	structure	1 hour 1µM	1 hour washout	effect	reversible
1	**Fragiformin C**	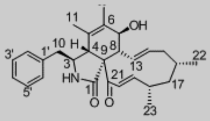	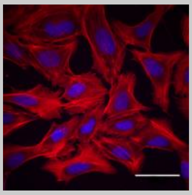	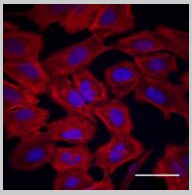	+	+/-
2	**Fragiformin D**	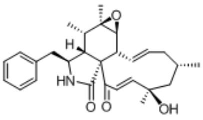	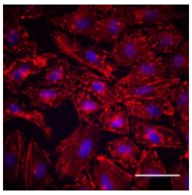	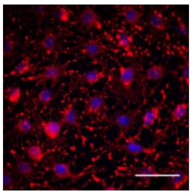	+++	-
3	**Saccalasin A**	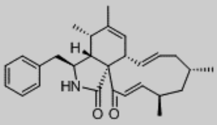	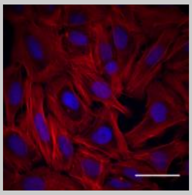	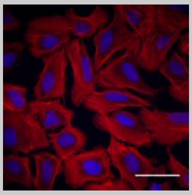	-	**n.d.**
4	**Cytochalasin B** <br>**(reference compound)**	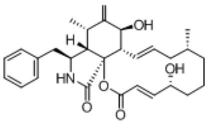	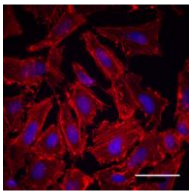	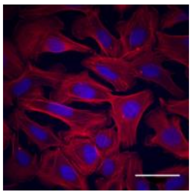	++	+
5	**Deoxaphomin**	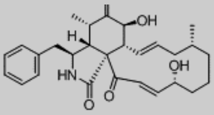	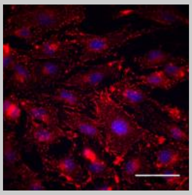	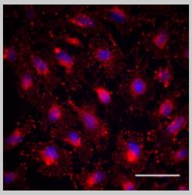	+++	-
6	**Cytochalasin D**	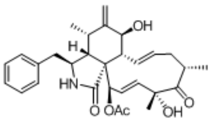	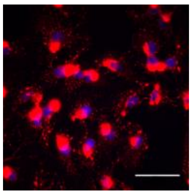	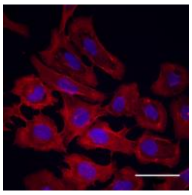	+++	+/-
7	**Cytochalasin F**	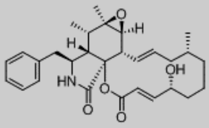	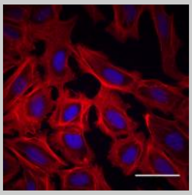	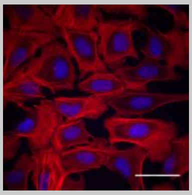	+	+

8	**Cytochalasin H**	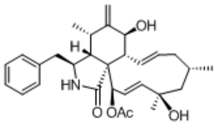	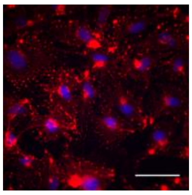	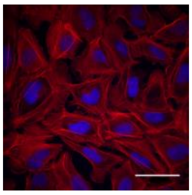	+++	+
9	**L-696,474**	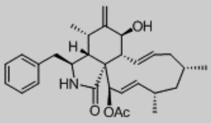	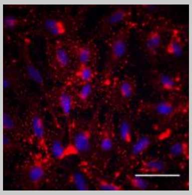	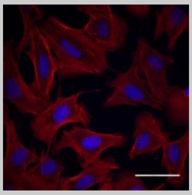	+++	+
10	**21-O-Deacyl-L-696,474**	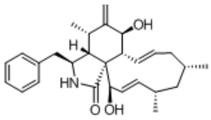	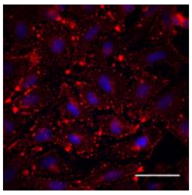	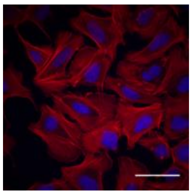	+++	+
11	**Cytochalasin Z2**	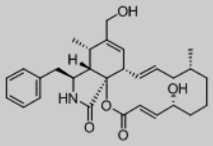	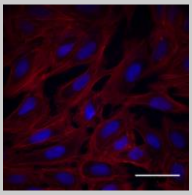	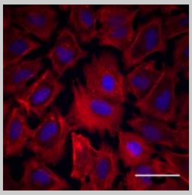	+	+
12	**12**	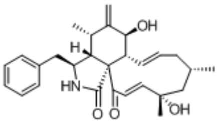	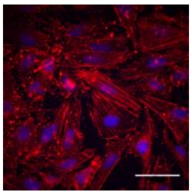	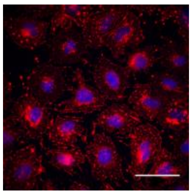	+++	-
13	**13**	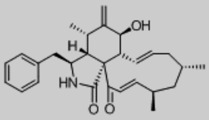	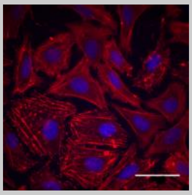	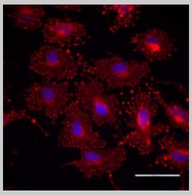	++	-
14	**14**	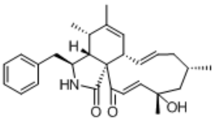	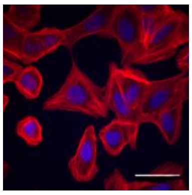	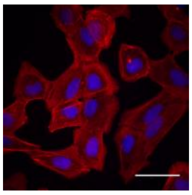	+	+/-
15	**15**	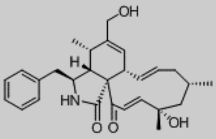	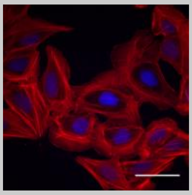	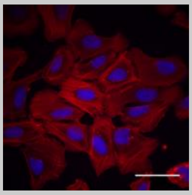	+	+/-

16	**16**	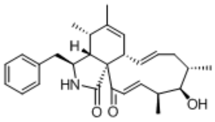	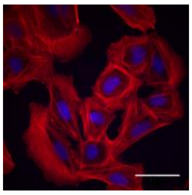	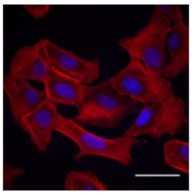	-	**n.d.**
17	**17**	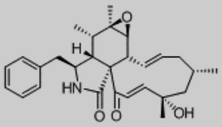	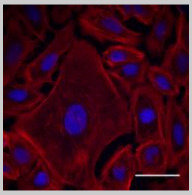	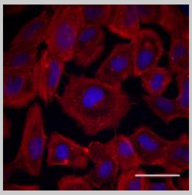	+	+/-
18	**19,20-Epoxycytochalasin C**	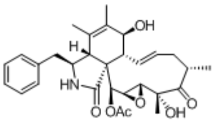	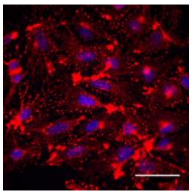	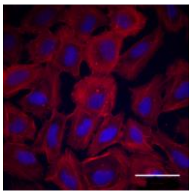	+++	+
19	**19,20-Epoxycytochalasin D**	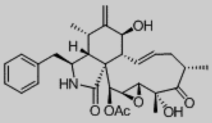	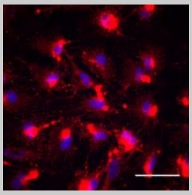	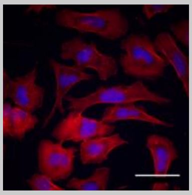	+++	+/-
20	**19,20-Epoxycytochalasin N**	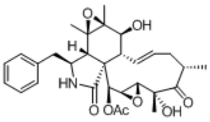	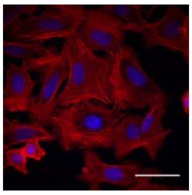	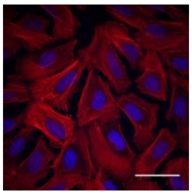	+	+
21	**18-Deoxy-19,20-Epoxycytochalasin D**	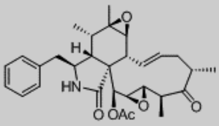	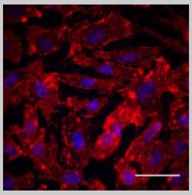	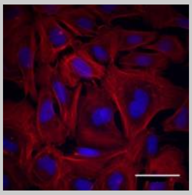	++	+
22	**Phenochalasin C**	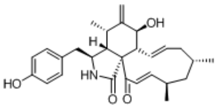	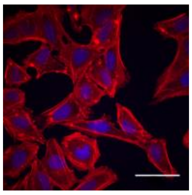	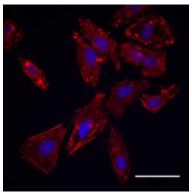	++	-
23	**Phenochalasin D**	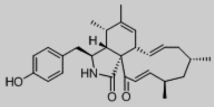	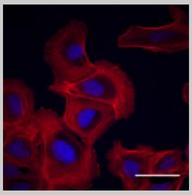	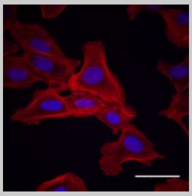	-	**n.d.**

24	Chaetoglobosin A	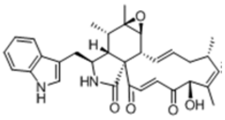	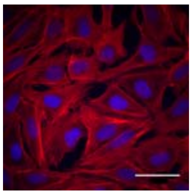	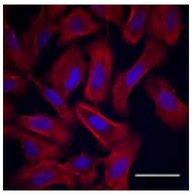	+	+
25	**Chaetoglobosin D**	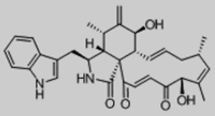	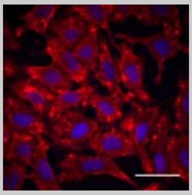	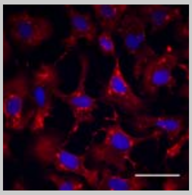	++	-
26	**DMSO**<br><br>**(vehicle control)**	-	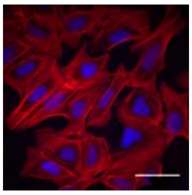	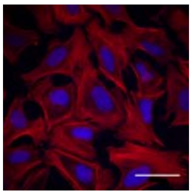	/	/

Table S1: Cytochalasan treatment of U2OS cells. U2OS cells [ATCC HTB-96] were cultivated in Dulbecco's modified minimum essential medium (DMEM, Life Technologies, Carlsbad, CA, USA) with 10% fetal bovine serum, 1% L-glutamine, 1% minimum essential medium nonessential amino acids (MEM NEAA) and 1% sodium pyruvate (Life technologies, Carlsbad, CA, USA) at 36°C and 5% CO_2. For cytochalasan treatment, cells were seeded on glass coverslips which in turn were placed in a six-well-plate. Prior to cell growth, the coverslips were coated with 25 µg/ml fibronectin in PBS for one hour. Cytochalasans were dissolved in DMSO (Carl Roth GmbH, Karlsruhe, Germany) for treatment. When the cells reached 80% confluency, cytochalasans were applied at a concentration of 1 and 5µg/ml for 1 h. Wash out experiments were conducted by exchanging the cytochalasan containing medium after incubation time with DMEM followed by incubation for another hour. As vehicle control, DMSO was used at the same concentrations (1 µg/ml, 5 µg/ml) as cytochalasans. After incubation, the cells were fixed by incubation with 4% paraformaldehyde (AppliChem, Darmstadt, Germany) in PBS for 20 min at room temperature. Fixed cells were washed with PBS and permeabilized with 0.1% Triton X-100 (Bio-Rad Laboratories, Hercules, CA, USA) in PBS for 1 min at room temperature. The cells were washed again thrice with PBS and afterwards stained using fluorescently labeled phalloidin ATTO 594 (ATTO-Tec, Siegen, Germany), diluted 1:200 in PBS. The coverslips were mounted in Prolong Diamond antifade mountant with DAPI (inVitrogen, Carlsbad, CA, USA) in order to stain the nucleus. Pictures of the cells were taken using a Axio Vert 135 TV inverse microscope with phase contrast and CoolSnap 4k camera (Zeiss, Oberkochen, Germany). Pictures were processed using the Image J software package (NIH, USA). The treatments were run in duplicates and the experiments repeated always with the same results.
